# The reliability paradox: Why robust cognitive tasks do not produce reliable individual differences

**DOI:** 10.3758/s13428-017-0935-1

**Published:** 2017-07-19

**Authors:** Craig Hedge, Georgina Powell, Petroc Sumner

**Affiliations:** School of Psychology, Cardiff University, Park Place, Cardiff, CF10 3AT UK

**Keywords:** Reliability, Individual differences, Reaction time, Difference scores, Response control

## Abstract

Individual differences in cognitive paradigms are increasingly employed to relate cognition to brain structure, chemistry, and function. However, such efforts are often unfruitful, even with the most well established tasks. Here we offer an explanation for failures in the application of robust cognitive paradigms to the study of individual differences. Experimental effects become well established – and thus those tasks become popular – when between-subject variability is low. However, low between-subject variability causes low reliability for individual differences, destroying replicable correlations with other factors and potentially undermining published conclusions drawn from correlational relationships. Though these statistical issues have a long history in psychology, they are widely overlooked in cognitive psychology and neuroscience today. In three studies, we assessed test-retest reliability of seven classic tasks: Eriksen Flanker, Stroop, stop-signal, go/no-go, Posner cueing, Navon, and Spatial-Numerical Association of Response Code (SNARC). Reliabilities ranged from 0 to .82, being surprisingly low for most tasks given their common use. As we predicted, this emerged from low variance between individuals rather than high measurement variance. In other words, the very reason such tasks produce robust and easily replicable experimental effects – low between-participant variability – makes their use as correlational tools problematic. We demonstrate that taking such reliability estimates into account has the potential to qualitatively change theoretical conclusions. The implications of our findings are that well-established approaches in experimental psychology and neuropsychology may not directly translate to the study of individual differences in brain structure, chemistry, and function, and alternative metrics may be required.

**Electronic supplementary material:**

The online version of this article (doi:10.3758/s13428-017-0935-1) contains supplementary material, which is available to authorized users.

*Individual differences have been an annoyance rather than a challenge to the experimenter. His goal is to control behavior*, *and variation within treatments is proof that he has not succeeded*… *For reasons both statistical and philosophical*, *error variance is to be reduced by any possible device*. (Cronbach, 1957, p. 674)

The discipline of psychology consists of two historically distinct approaches to the understanding of human behavior: the correlational approach and the experimental approach (Cronbach, 1957). The division between experimental and correlational approaches was highlighted as a failing by some theorists (Cronbach, 1957; Hull, 1945), whilst others suggest that it may be the inevitable consequence of fundamentally different levels of explanation (Borsboom, Kievit, Cervone, & Hood, 2009). The correlational, or individual differences, approach examines factors that distinguish between individuals within a population (i.e., between-subject variance). Alternatively, the experimental approach aims to precisely characterize a cognitive mechanism based on the typical or average response to a manipulation of environmental variables (i.e., within-subject variance). Cronbach (1957) called for an integration between the disciplines, with the view that a mature science of human behavior and brain function would consist of frameworks accounting for both inter- and intra-individual variation. Whilst a full integration is far from being realized, it is becoming increasingly common to see examinations of the neural, genetic, and behavioral correlates of performance on tasks with their origins in experimental research (e.g., Chen et al., 2015; Crosbie et al., 2013; Forstmann et al., 2012; Marhe, Luijten, van de Wetering, Smits, & Franken, 2013; L. Sharma, Markon, & Clark, 2014; Sumner, Edden, Bompas, Evans, & Singh, 2010).

Such integration is not without obstacles (e.g., Boy & Sumner, 2014). Here, we highlight a general methodological consequence of the historical divide between experimental and correlational research. Specifically we ask whether tasks with proven pedigree as “reliable” workhorses in the tradition of experimental research are inevitably unsuitable for correlational research, where “reliable” means something different. This issue is likely to be ubiquitous across all domains where robust experimental tasks have been drawn into correlational studies, under the implicit assumption that a robust experimental effect will serve well as an objective measure of individual variation. This has occurred, for example, to examine individual differences in cognitive function, brain structure, and genetic risk factors in neuropsychological conditions (e.g.. Barch, Carter, & Comm, 2008), or where individual difference analyses are performed as supplementary analyses in within-subject studies (c.f. Yarkoni & Braver, 2010). Many of the issues we discuss reflect long-recognized tensions in psychological measurement (Cronbach & Furby, 1970; Lord, 1956), though they are rarely discussed in contemporary literature. The consequences of this are that researchers often encounter difficulty when trying to translate state-of-the art experimental methods to studying individual differences (e.g., Ross, Richler, & Gauthier, 2015). By elucidating these issues in tasks used prominently in both experimental and correlational contexts, we hope to aid researchers looking to examine behavior from both perspectives.

## The reliability of experimental effects

### Different meanings of reliability

For experiments, a “reliable” effect is one that nearly always replicates, one that is shown by most participants in any study and produces consistent effect sizes. For example, in the recent “Many labs 3” project (Ebersole et al., 2016), which examined whether effects could be reproduced when the same procedure was run in multiple labs, the Stroop effect was replicated in 100% of attempts, compared to much lower rates for most effects tested.

In the context of correlational research, reliability refers to the extent to which a measure consistently *ranks individuals*. This meaning of reliability is a fundamental consideration for individual differences research because the reliability of two measures limits the correlation that can be observed between them (Nunnally, 1970; Spearman, 1904). Classical test theory assumes that individuals have some “true” value on the dimension of interest, and the measurements we observe reflect their true score plus measurement error (Novick, 1966). In practice, we do not know an individual’s true score, thus, reliability depends on the ability to consistently rank individuals at two or more time points. Reliability is typically assessed with statistics like the IntraClass Correlation (ICC), which takes the form:

$$ I C C=\frac{Variance\kern0.5em  between\kern0.5em  individuals}{Variance\kern0.5em  between\kern0.5em  individuals\kern0.5em + Error\kern0.5em  variance\kern0.5em + Variance\kern0.5em  between\kern0.5em  sessions} $$

1Here, variance between sessions corresponds to systematic changes between sessions across the sample. Error variance corresponds to non-systematic changes between individuals’ scores between sessions, i.e. the score for some individuals increases, while it decreases for others. Clearly, reliability decreases with higher measurement error, whilst holding variance between participants constant. Critically, *reliability also decreases for smaller between*-*participant variance*, whilst holding error variance constant. In other words, for two measures with identical “measurement error,” there will be lower reliability for the measure with more homogeneity. Measures with poor reliability are ill-suited to correlational research, as the ability to detect relationships with other constructs will be compromised by the inability to effectively distinguish between individuals on that dimension (Spearman, 1910).

In contrast to the requirements for individual differences, homogeneity is the ideal for experimental research. Whereas variance between individuals is the numerator in the ICC formula above, it appears as the denominator in the *t*-test (i.e., the standard error of the mean). For an experimental task to produce robust and replicable results, it is disadvantageous for there to be large variation in the within-subject effect. Interestingly, it is possible for us to be perfectly aware of this for statistical calculations, without realising (as we previously didn't) that the meanings of a “reliable” task for experimental and correlational research are not only different, but can be opposite in this critical sense.

## Present study

The issues we discuss have broad implications for cognitive psychology and cognitive neuroscience. Recent reviews have highlighted the potential for individual differences approaches to advance our understanding of the relationship between brain structure and function (Kanai & Rees, 2011). The way in which we measure and conceptualize cognitive processes has largely been built on within-subject paradigms, though their strengths in experimental contexts may make these paradigms sub-optimal for individual differences. Here, in three studies, we evaluate the re-test reliability of seven commonly used and robust tasks, spanning the domains of cognitive control, attention, processing style, and numerical-spatial associations. In doing so, we not only provide sorely needed information on these measures, but also evaluate the relationship between robust experimental paradigms and reliable individual differences in real data using cohort sizes and trial numbers similar to, or greater than, most imaging studies. In addition, we illustrate how taking the reliability of these measures into account has the power to change the conclusions we draw from statistical tests.

First, we examined the reliability of the Eriksen flanker task, Stroop task, go/no-go task, and the stop-signal task, which we then replicated in Study 2. These tasks are all considered to be measures of impulsivity, response inhibition or executive functioning (Friedman & Miyake, 2004; Stahl et al., 2014). In Study 3, we examined the Posner cueing task (Posner, 1980), the Navon task (Navon, 1977), and a spatial-numerical association of response codes (SNARC) effect paradigm (Dehaene, Bossini, & Giraux, 1993). These tasks are used to measure the constructs of attentional orienting, perceptual processing style, and the automatic association between magnitude and space (i.e., the “mental number line”), respectively. These tasks were selected because they were all originally developed in experimental contexts, and we believed they would be familiar to most readers. Further, all these tasks have since been used in the context of individual differences, and their underlying neural correlates. A Google Scholar search for the term “individual differences” within articles citing the original papers for each task produces at least 400 citations for each. For conciseness, we combine the reporting of our methods and results across all studies.

## Method

### Participants

Participants in Study 1 were 50 (three male) undergraduate students aged 18–21 years (M = 19.5 years, SD=0.9). Participants in Study 2 were 62 (12 male) undergraduate students aged 18–47 years (M = 20.5 years, SD=4.98). Participants in Study 3 were 42 (five male) undergraduate students aged 18–40 years (M = 20.4 years, SD=3.5). All participants gave informed written consent prior to participation in accordance with the revized Declaration of Helsinki (2013), and the experiments were approved by the local Ethics Committee.

### Design and procedure

Participants completed the tasks (four in Studies 1 and 2, three in Study 3) in each of two 90-min sessions taking place 3 weeks apart, at the same time of day. Seven participants in Study 1 and five participants in Study 2 were unable to attend their second session exactly 3 weeks later, and were rescheduled to between 20 and 28 days following their first session. Each participant completed the tasks in the same order in both of their sessions (in order not to introduce between-session variance associated with order), and the order of tasks was counterbalanced across participants using a Latin square. Though counterbalancing is common practice in experimental studies, it is often preferable to administer tasks in a fixed order when correlating variables (though not all do, see e.g., Aichert et al., 2012; Wöstmann et al., 2013). However, our primary focus here was the re-test reliability of the tasks, and a fixed order could cause one task to appear more reliable than another due to presentation order rather than the task itself.

Following completion of the tasks, participants completed the UPPS-P impulsive behavior scale (Lynam, Smith, Whiteside, & Cyders, 2006; Whiteside & Lynam, 2001), which we commonly administer in our lab. We include reliability information for the UPPS-P components as a reference for the levels of reliability attainable in our sample with a measure constructed for the purpose of measuring individual differences.

**Fig. 1:**
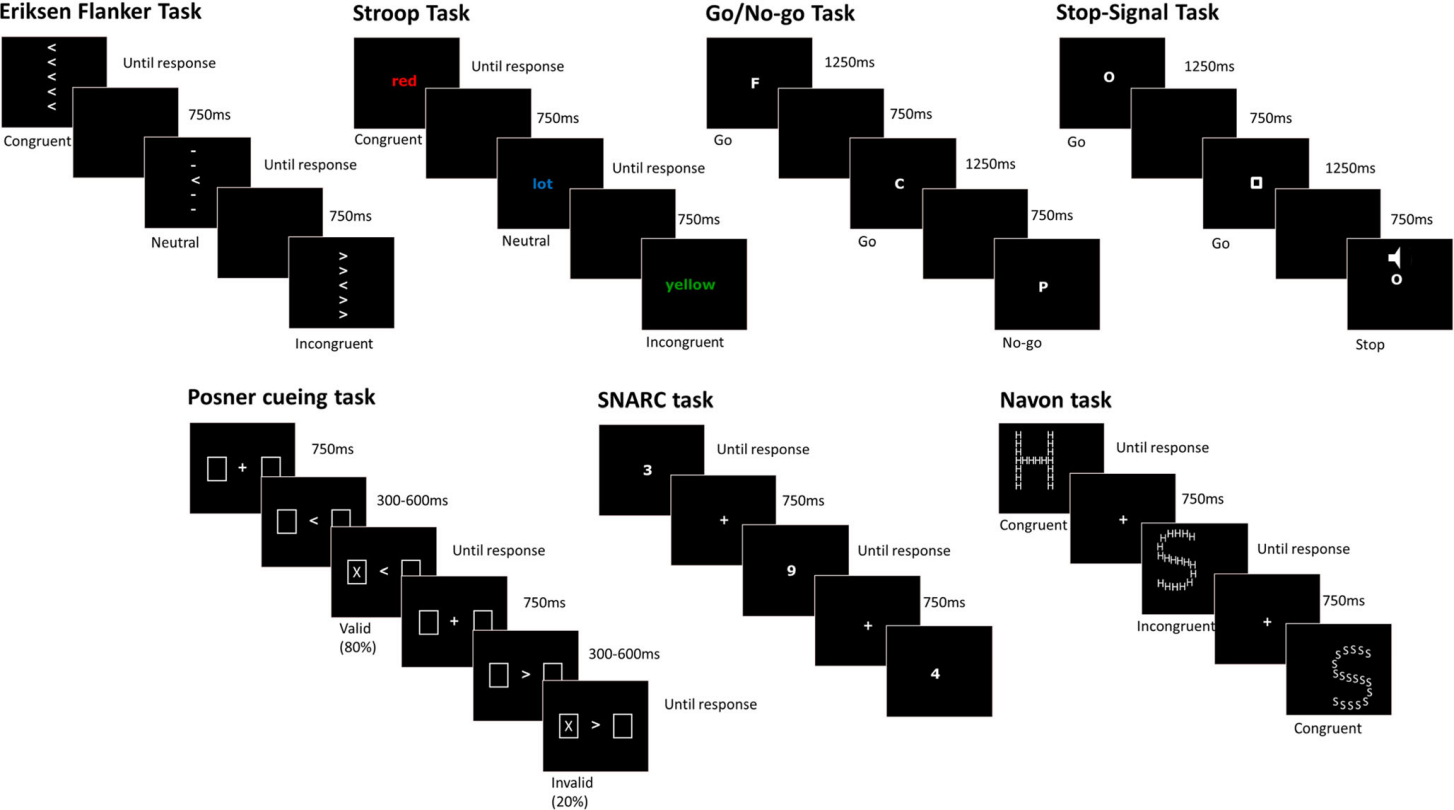
Schematic representation of tasks used and their conditions. Studies 1 and 2 featured the flanker, Stroop, go/no-go and stop-signal tasks. Study 3 featured the Posner cueing, SNARC and Navon tasks. Trials were presented intermixed in a randomized order. In the Go/no-go and Stop-signal tasks, visual stimuli were presented for a fixed duration of 1,250 ms (c.f. Verbruggen et al., 2008). In all other tasks, stimuli were presented until a response was given. An Inter-Stimulus Interval (ISI) of 750 ms was used in all tasks. Stimuli sizes are enlarged for illustration

Participants were tested in groups of up to nine, at separate stations in a multi-station lab, separated by dividers. The experimenter was present throughout the session to monitor compliance with instructions. Participants were instructed to be as fast and as accurate as possible in all tasks, and were given written and verbal instructions before each task. Each task in Studies 1 and 2 consisted of five blocks of approximately 4 min each, and participants received feedback about their average reaction times (RTs) and error rates after each block. The tasks in Study 3 consisted of four blocks. Figure 1 displays the format of the tasks used. The stop-signal task was implemented using STOP-IT (Verbruggen, Logan, & Stevens, 2008), all other tasks were implemented in PsychoPy (Peirce, 2007, 2013). An Inter-Stimulus Interval (ISI) of 750 ms was used for all tasks.

#### Eriksen flanker task

Participants responded to the direction of a centrally presented arrow (left or right) using the \ and / keys. On each trial, the central arrow (1 cm × 1 cm) was flanked above and below by two other symbols separated by 0.75 cm (see, e.g., Boy, Husain, & Sumner, 2010; White, Ratcliff, & Starns, 2011). Flanking stimuli were arrows pointing in the same direction as the central arrow (congruent condition), straight lines (neutral condition), or arrows pointing in the opposite direction to the central arrow (congruent condition). Stimuli were presented until a response was given. Participants completed 240 trials in each condition (720 in total). The primary indices of control are the RT cost (incongruent RT – congruent RT) and error rate cost (congruent errors – incongruent errors).

#### Stroop task

Participants responded to the color of a centrally presented word (Arial, font size 70), which could be red (z key), blue (x key), green (n key), or yellow (m key). (c.f. Ilan & Polich, 1999; Macleod, 1991; D. Sharma & McKenna, 1998). The word could be the same as the font color (congruent condition), one of four non-color words (lot, ship, cross, advice) taken from Friedman and Miyake (2004) matched for length and frequency (neutral condition), or a color word corresponding to one of the other response options (incongruent). Stimuli were presented until a response was given. Participants completed 240 trials in each condition (720 in total). The primary indices of control are the RT cost (incongruent RT – congruent RT) and error rate cost (congruent errors – incongruent errors).

#### Go/No-go task

Participants were presented with a series of letters (Arial, font size 70) in the center of the screen. Each block consisted of four letters, presented with equal probability. Participants were instructed to respond with the space bar to three of the four letters (go trials), and to refrain from responding if the fourth letter appeared (no-go trials). The response rule was presented to participants at the beginning of each block, and displayed at the bottom of the screen throughout the block to reduce memory demands. A new set of letters was used for each block, to lessen the impact of learned, automatic associations (c.f. Verbruggen & Logan, 2008). Stimuli were presented for a fixed duration of 1,250 ms. Participants completed 600 trials in total (75% go). The primary measures are commission errors (responses to no-go stimuli), omission errors (non-responses to go stimuli), and RT to go stimuli.

#### Stop-signal task

Participants were instructed to respond to the identity of a centrally presented stimulus (square or circle: 1.6 cm × 1.6 cm) using the \ and / keys. On 25% of trials (stop trials), participants heard a tone through a set of headphones that indicated that they should withhold their response on that trial. The tone was initially presented 250 ms after the visual stimulus appeared, and was adjusted using a tracking procedure by which the latency increased by 50 ms following a successfully withheld response, and decreased by 50 ms following a failure to withhold a response. The latency of the tone is referred to as the Stop-Signal Delay (SSD). Stimuli were presented for a fixed duration of 1,250ms. Participants completed 600 trials in total (75% go). The primary measures are Stop-Signal Reaction Time (SSRT), and go RT. There are two common methods of calculating SSRT: the mean method (SSRTm) and the integration method (SSRTi; Logan, 1981; Logan & Cowan, 1984). The mean method consists of subtracting the participant’s mean SSD from their mean go RT. In the integration method, instead of the mean go RT, the mean SSD is subtracted from the nth fastest RT, where n corresponds to the percentage of stop trials on which participants failed to inhibit their responses. For example, if a participant responded on 60% of stop trials, the 60th percentile of their RT distribution is subtracted from the mean SSD. Accurate estimation of SSRT using the mean method relies upon the tracking procedure converging on successful stopping on 50% of stop trials. It has been argued that the integration method should be favoured when this assumption is not met, for example, if participants strategically adjust their responses by slowing down over the course of the session (Verbruggen, Chambers, & Logan, 2013). We report the reliabilities of both methods here, but restrict subsequent analyses to only the recommended integration method.

#### Posner cueing task

At the start of each trial, participants viewed two boxes (6 cm × 6 cm), located 7.5 cm from a central fixation point to the inside edge. An arrow cue (2 cm × 1.5 cm) appeared in the center of the screen directing participants’ attention to either the left or the right box. After a stimulus onset asynchrony (SOA) of 300, 400, 500, or 600 ms, an X (2 cm × 2 cm) then appeared in the left or right box. Participants were instructed to respond as quickly as possible with the space bar to the critical stimulus, but to not respond before it appeared. The cue correctly predicted the location of the stimulus on 80% of trials, and participants were instructed of this probability beforehand. The SOAs were chosen to make the onset of the stimulus unpredictable, and previous research has shown that the cueing benefit peaks at approximately 300 ms and is consistent throughout this range of SOAs (Cheal & Lyon, 1991; Muller & Rabbitt, 1989). If participants responded before the stimulus appeared, they were given feedback lasting 2,500 ms instructing them not to respond prematurely. Participants were instructed to maintain their fixation on the central fixation point/cue. Participants completed 640 trials (128 invalid) in total. The key measure of interest is the difference in RTs to stimuli following valid compared to invalid cues.

#### Spatial-numerical association of response codes (SNARC) task

Participants were required to determine whether a centrally presented white digit (1–9, excluding 5; Arial, font size 70) was greater or less than five. Before each block, participants were instructed that they were to respond either such that Z corresponded to digits less than five and M digits greater than five, or vice versa. This rule alternated across blocks, with the first block being counter-balanced across participants, and participants receiving consistent order in both of their sessions. As in previous studies (e.g., Rusconi, Dervinis, Verbruggen, & Chambers, 2013), eight “buffer” trials were presented at the start of each block to accommodate the change in response rules. These buffer trials were subsequently discarded for analysis. Participants were also presented with feedback if they gave an incorrect response, lasting 1,000 ms. Participants completed 640 trials in total (320 with each mapping), not including buffer trials. The SNARC effect is the key variable of interest, which is calculated as the difference between RTs and error rates on trials in which the required response aligns with the relative magnitude of the stimulus compared to when they are misaligned. Participants are expected to respond more quickly to smaller numbers with the left hand and larger numbers with the right.

#### Navon task

Participants were presented with composite letter stimuli; large “H” or “S” characters (3 cm × 4.5 cm) comprised of smaller “S” or “H” (0.4 cm × 0.7 cm) characters. Stimuli could either be consistent, in which the same character appeared at the global and local levels, or inconsistent (e.g., a large H composed of smaller S characters). Stimuli were presented at one of four possible locations and remained on screen until a response was given. The stimuli were presented 0.5 cm above or below and 2 cm to the left or right of fixation. Before each block, participants were instructed that they were to respond to either the global or local character. The response rule alternated across blocks, and was counter-balanced, as with the SNARC task. Further, as with the SNARC task, participants were presented with eight buffer trials, and feedback to incorrect response. Participants completed 640 trials in total (320 per mapping, of which 160 each were consistent and inconsistent). We derived five effects of interest from this task. We calculated the difference between congruent RTs for responses to global versus local stimuli as an indication of participants’ bias towards global or local processing (with healthy participants typically showing a global bias). Further, interference effects in both errors and RTs (Incongruent - congruent) can be derived for global and local stimuli separately.

#### UPPS-P impulsive behavior scale

The UPPS-P is a 59-item questionnaire that measures five components of impulsivity: negative urgency, premeditation, perseverance, sensation seeking, and positive urgency (Lynam et al., 2006; Whiteside & Lynam, 2001).

## Data analysis

Data were not included if participants did not return for the follow-up session (3,2,2 for the three studies respectively). Participants' data were not analysed for a given task if they show very low compliance, defined as: accuracy below 60% in either session for overall performance in the flanker, Stroop, Navon, and SNARC tasks, responses to go stimuli in the go/no-go task, discrimination performance on go trials in the stop-signal task. For the Posner task, participants were also required to have anticipatory response rates (i.e., responding before the stimulus appears) of less than 10%. For the stop signal task, participants’ data were not included if their data produced a negative SSRT, or if they responded on more than 90% of stop-signal trials in either session, as an SSRT could not be meaningfully calculated. A participant’s data was removed entirely if they fell below these criteria for two or more tasks within a single session, otherwise data were only excluded for the individual task. After these exclusions, 47 and 57 participants remained for the flanker and go/no-go tasks in Study 1 and 2, respectively, 47 and 56 in the Stroop task, and 45 and 54 in the stop-signal task. All participants met the inclusion criteria in Study 3. The calculation of mean RTs excluded RTs below 100 ms and greater than three times the each individual’s median absolute deviation (Hampel, 1974; Leys, Ley, Klein, Bernard, & Licata, 2013).

Reliabilities were calculated using Intraclass Correlation Coefficients (ICC) using a two-way random effects model for absolute agreement. In the commonly cited Shrout and Fleiss (1979; see also McGraw & Wong, 1996) nomenclature, this corresponds to ICC (2,1). This form of the ICC is sensitive to differences between session means. In Supplementary Material A, we perform further analyses to account for potential outliers and distributional assumptions. The choice of statistic does not affect our conclusions. We report reliabilities separately for Studies 1 and 2 in the main text so that consistency across samples can be observed. We combine the studies in supplementary analyses.

As both measurement error and between-participant variability are important for the interpretation of reliability, we also report the standard error of measurement (SEM) for each variable. The SEM is the square root of the error variance term in the ICC calculation and reflects the 68% confidence interval around an individual’s observed score.

Summary level data, as well as the raw data for our behavioral tasks, are available on the Open Science Framework (https://osf.io/cwzds/)

## Results

### Task performance

#### Studies 1 and 2

**Fig. 2:**
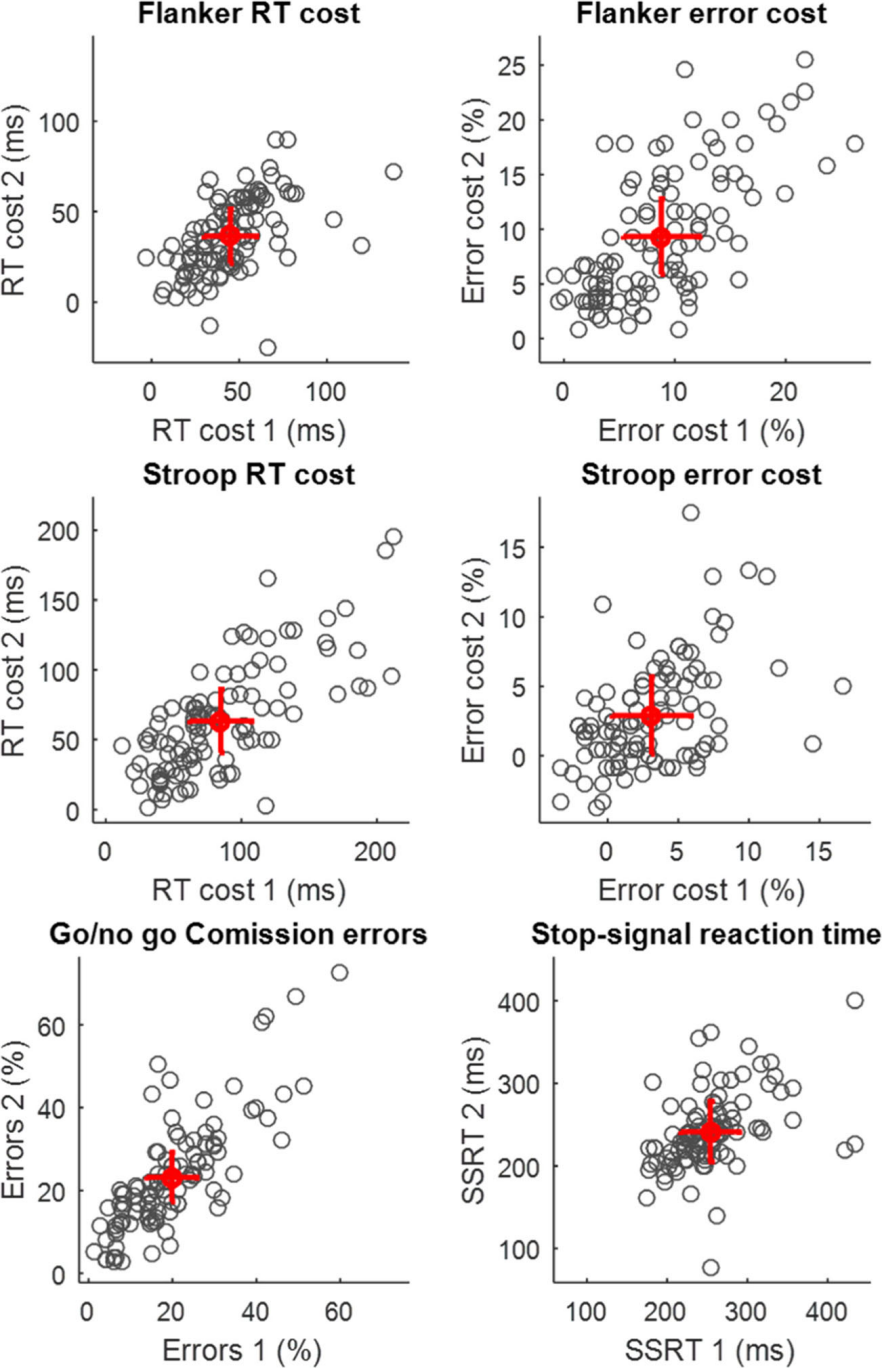
Reliability of key measures from Studies 1 and 2 combined (Total N=99–104). Red marker indicates mean group performance from sessions 1 and 2. Error bars show ± 1 standard error of measurement (SEM). The SEM is the square root of the error variance term calculated from the intraclass correlation, and can be interpreted as the 68% confidence interval for an individual’s data point. A large SEM relative to the between-subject variance contributes to poor reliability

A full report of the descriptive statistics for each measure can be seen in Supplementary Material B. All expected experimental effects were observed, and means and standard deviations for RTs and error rates for all tasks were comparable to samples from the general population reported in the literature (see Supplementary Material C). Thus, despite a possible expectation that students would show restricted variance, our sample was not consistently more or less variable than samples taken from the general population. Scatter plots for the key measures are shown in Fig. 2.

#### Study 3

**Fig. 3:**
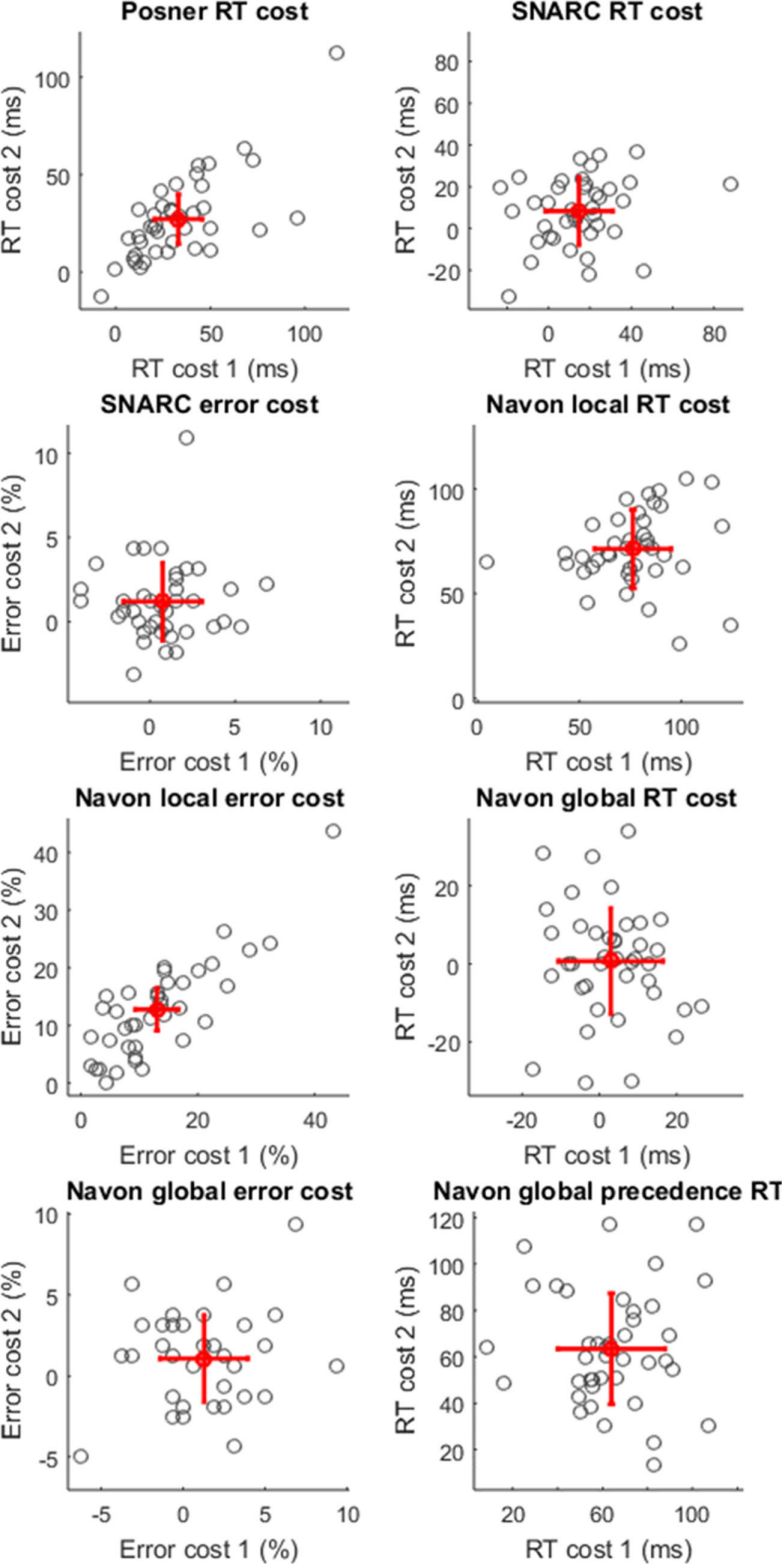
Reliability of key measures from Study 3 (N=40). Red marker indicates mean group performance from sessions 1 and 2. Error bars show ± 1 standard error of measurement. *RT* reaction time, *SNARC* Spatial-Numerical Association of Response Code

Again, performance was comparable to previous reports in the literature (Navon, 1977; Posner, 1980; Rusconi et al., 2013). As in Navon’s original study, the conflict effect in the RTs did not reach significance when participants were instructed to respond to the global characters and ignore the local characters – presumably reflecting the preferential processing of global features. Scatter plots for the key measures are shown in Fig. 3.

## Task reliabilities

### Studies 1 and 2

None of the behavioral measures in Studies 1 and 2 (see Table 1) exceeded reliabilities of .8, typically considered excellent or of a clinically required standard (Cicchetti & Sparrow, 1981; Fleiss, 1981; Landis & Koch, 1977). Two indices of response control exceeded a standard of good/substantial reliability (.6) in both sessions: the Stroop RT cost (ICCs of .6 and .66 in Studies 1 and 2 respectively) and commission errors on the go/no-go task (ICC = .76 in both studies). The reliability of the RT cost scores, calculated by taking the difference between congruent and incongruent conditions for example, are generally lower than their components, and we examine reasons for this below. For example, the flanker RT cost in Study 1 has a reliably of .4, whereas the RTs for congruent and incongruent trials have reliabilities of .74 and .66 respectively. This is despite the flanker RT cost having a relatively low SEM of 15 ms. Thus, measurement error alone does not predict reliability. The scatter plots in Fig. 2 show the SEMs for the critical measures to show the size of the error relative to the variance in the data.

The results for the stop signal task warrant expansion. Large SEMs were observed for Go RT and mean SSD in Study 1. We suspect that this is due to proactive slowing in a subset of participants in one session, who did not strategically adjust their responses in the same way in the other session. However, despite a reduced SEM and higher reliability for go RTs in Study 2, the reliability of SSRT did not increase. Though the integration method of calculating SSRT was shown by Verbruggen et al. (2013) to be robust against gradual slowing within a session, it will remain sensitive to more substantial strategic changes between sessions (c.f., Leotti & Wager, 2010). Adopting a more conservative exclusion criterion did not improve upon the reliability estimates for SSRTs (see Supplementary Material A).

**Table 1:** Intraclass correlations (ICCs) and standard errors of measurement (SEMs) for Studies 1 and 2. SEMs are in the measure’s original units (ms or % correct). Primary indices of response control are highlighted in bold; 95% confidence intervals in parentheses. Typical interpretations of ICC values are: excellent (.8), good/substantial (.6), and moderate (.4) levels of reliability (Cicchetti & Sparrow, 1981; Fleiss, 1981; Landis & Koch, 1977)

Task	Measure	ICCs		SEMs	
		Study 1	Study 2	Study 1	Study 2
Flanker task	Congruent RT	.74 (.52–.86)	.69 (.40 –.83)	24 (20–30)	20 (17–24)
	Neutral RT	.73 (.48–.86)	.61 (.32–.78)	23 (19–29)	21 (18–26)
	Incongruent RT	.66 (.36–.81)	.62 (.31–.79)	32 (27–40)	28 (24–35)
	**RT cost**	.**40** (.**12**–.**61**)	.**57** (.**36**–.**72**)	**15** (**13**–**19**)	**15** (**13**–**18**)
	Congruent errors	.46 (.20–.66)	.37 (.13–.58)	4.78 (3.97–6.0)	5.24 (4.43–6.43)
	Neutral errors	.45 (.19–.65)	.39 (.14–.59)	4.95 (4.11–6.22)	5.16 (4.36–6.33)
	Incongruent errors	.71 (.54–.83)	.58 (.34–.74)	4.67 (3.88–5.86)	5.76 (4.86–7.07)
	**Error cost**	.**58** (.**35**–.**74**)	.**72** (.**57**–.**83**)	**3.77** (**3.14**–**4.74**)	**3.12** (**2.64**–**3.83**)
Stroop task	Congruent RT	.77 (.49–.88)	.72 (.49–.84)	33 (27 –41)	31 (26–38)
	Neutral RT	.74 (.36–.88)	.73 (.45–.86)	34 (28–43)	34 (28–41)
	Incongruent RT	.67 (.25–.85)	.70 (.10–.88)	42 (35–52)	33 (28–40)
	**RT cost**	.**60** (.**31**–.**78**)	.**66** (.**26**–.**83**)	**21** (**17**–**26**)	**24** (**20**–**29**)
	Congruent errors	.36 (.10–.58)	.42 (.16–.62)	3.35 (2.78–4.20)	3.02 (2.55–3.71)
	Neutral errors	.45 (.19–.65)	.51 (.25–.69)	3.52 (2.92–4.42)	3.17 (2.67–3.89)
	Incongruent errors	.62 (.40–.77)	.39 (.15–.59)	3.78 (3.14–4.75)	3.89 (3.28–4.78)
	**Error cost**	.**48** (.**23**–.**67**)	.**44** (.**20**–.**63**)	**3.13** (**2.60** –**3.94**)	**2.45** (**2.07**–**3.02**)
Go/No-go task	Go RT	.74 (.58–.85)	.63 (.44–.77)	31 (25–38)	37 (31–46)
	**Commission errors**	.**76** (.**58**–.**87**)	.**76** (.**60**–.**86**)	**5.36** (**4.45**–**6.73**)	**6.46** (**5.46**–**7.93**)
	Omission errors	.69 (.51–.82)	.42 (.19–.61)	1.52 (1.27–1.91)	3.73 (3.15–4.57)
Stop-signal task	Go RT	.35 (.08–.57)	.57 (.28–.75)	107 (88–135)	57 (48–70)
	Mean SSD	.34 (.07–.57)	.54 (.32–.70 )	127 (105–161)	71 (60–88)
	**SSRT mean**	.**47** (.**21**–.**67**)	.**43** (.**19**–.**62**)	**32** (**27**–**41**)	**28** (**24**–**35**)
	**SSRT integration**	.**36** (.**08**–.**59**)	.**49** (.**26**–.**66**)	**39** (**32**–**49**)	**35** (**29**–**43**)
UPPS-P	Negative U.	.72 (.54–.83)	.73 (.58–.83)	.30 (.25–.38)	.29 (.25–.36)
	Premeditation	.70 (.51–.82)	.85 (.75–.91)	.26 (.21–.32)	.18 (.15–.22)
	Perseverance	.73 (.57–.84)	.78 (.65–.86)	.29 (.24–.36)	.21 (.18–.26)
	Sensation Seek.	.87 (.78–.93)	.89 (.82–.94)	.24 (.20–.30)	.21 (.18–.26)
	Positive U.	.80 (.66–.88)	.81 (.70–.88)	.25 (.21–.32)	.29 (.24–.36)

*RT* reaction time, *SSD* Stop-Signal Delay, *SSRT* Stop-Signal Reaction Time, *UPPS*-*P* impulsive behavior scale

### Study 3 (see Table 2)

Only one behavioral measure had a reliability in the nominally excellent range (.82): the conflict effect when responding to local characters in the Navon task. An influential data point (an error cost of 43% in both sessions) contributed to this, though the measure still shows good reliability (.74) if this individual is excluded.

**Table 2:** Intraclass correlations (ICCs) and standard errors of measurement (SEMs) for Study 3. SEMs are in the measure’s original units (ms or % correct). Primary variables of interest are highlighted in bold; 95% confidence intervals in parentheses. Typical interpretations of ICC values are: excellent (.8), good/substantial (.6), and moderate (.4) levels of reliability (Cicchetti & Sparrow, 1981; Fleiss, 1981; Landis & Koch, 1977). The Global precedence effect was calculated as local congruent RT – global congruent RT

	Measure	ICC	SEM
Posner task	Valid RT	.80 (.61–.90)	16 (13–20)
	Invalid RT	.79 (.56–.89)	21 (18–28)
	**Cueing effect**	.**70** (.**50**–.**83**)	**13** (**10**–**16**)
SNARC task	Congruent RT	.69 (.49–.82)	29 (24–37)
	Incongruent RT	.74 (.56–.86)	26 (21–33)
	**SNARC effect RT**	.**22** (**0**–.**49**)	**16** (**13**–**21**)
	Congruent errors	.67(.45–.81)	2.04 (1.67–2.62)
	Incongruent errors	.58 (.33–.75)	2.66 (2.18–3.42)
	**SNARC effect errors**	.**03** (**0**–.**34**)	**2.30** (**1.88**–**2.95**)
Navon task	Local congruent RT	.69 (.49–.83)	29 (24–38)
	Local incongruent RT	.68 (.45–.83)	30 (24–38)
	**Local RT cost**	.**14** (**0**–.**43**)	**19** (**15**–**24**)
	Local congruent errors	.56 (.30–.74)	1.23 (1.01–1.58)
	Local incongruent errors	.80 (.65–.89)	4.25 (3.48–5.46)
	**Local error cost**	.**82** (.**69**–.**90**)	**3.68** (**3.01**–**4.72**)
	Global congruent RT	.63 (.40–.78)	34 (28–43)
	Global incongruent RT	.70 (.50–.83)	30 (25–39)
	**Global RT cost**	**0** (**0**–.**18**)	**14** (**11**–**17**)
	Global congruent errors	.60 (.36–.76)	2.22 (1.82–2.86)
	Global incongruent errors	.71 (.51–.84)	1.96 (1.61–2.52)
	**Global error cost**	.**17** (**0**–.**46**)	**2.67** (**2.19**–**3.43**)
	**Global precedence effect** (**RT**)	**0** (**0**–.**29**)	**24** (**20**–**31**)
UPPS-P	Negative U.	.78 (.63–.88)	0.22 (0.18–0.29)
	Premeditation	.88 (.78–.93)	0.14 (0.12–0.18)
	Perseverance	.90 (.81–.94)	0.18 (0.14–0.23)
	Sensation Seek.	.91 (.83–.95)	0.16 (0.13–0.20)
	Positive U.	.85 (.67–.93)	0.20 (0.17–0.26)

*RT* reaction time, *UPPS*-*P* impulsive behavior scale, *SNARC* Spatial-Numerical Association of Response Code

The reliability of the Posner cueing effect was good (.7), though also influenced by an outlying data point (ICC = .56 if excluded). The reliabilities for all other behavioral effects of interest were poor (ICCs <.25).

### How many trials should be administered?

We found that the literature on these seven tasks also lacks information to guide researchers on how many trials to run, and different studies can choose very different numbers without any explicit discussion or justification. For those interested in the use of these tasks for individual differences, we provide information on the relationship between reliability and trial numbers in Supplementary Material D.

## What happens to variance in within-subject effects?

**Fig. 4:**
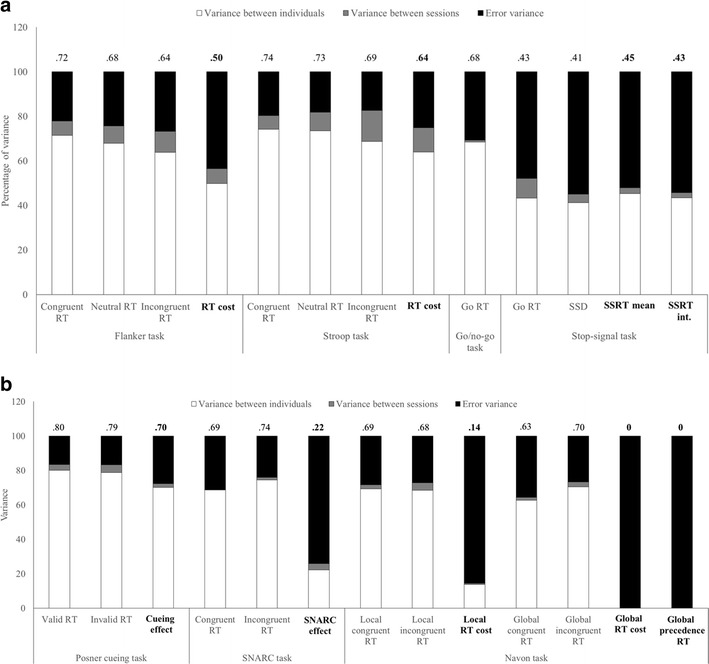
Relative size of variance components for reaction time (RT) measures in Studies 1 and 2 (A: Total N=99–104) and Study 3 (B: N=40). The size of the bar is normalized for the total amount of variance in the measure (see Supplementary Material E), and subdivided into variance accounted for by differences between participants (white), variance accounted for by differences between sessions (e.g., practice effects, gray), and error variance (black). The intraclass correlation (ICC) reflects the proportion of the total variance attributed to variance between individuals, and is printed above each bar. *SSD* Stop-Signal Delay,*SSRT* Stop-Signal Reaction Time, *SNARC* Spatial-Numerical Association of Response Code

The relationship between reliability and the sources of variance in the RT measures is shown in Fig. 4, which plots the three components of variance from which the ICC is calculated. Each bar decomposes the relative variance accounted for by differences between participants (white), differences between sessions (e.g., practice effects, gray), and error variance (black). Correlational research (and the ICC) relies on the proportion of variance accounted for by individual differences, and the standard subtractions (e.g., to calculate the Stroop RT cost) do not improve this signal-to-noise ratio – if anything, it is reduced, explaining why difference scores are generally lower in reliability than their components. The equivalent plot for errors can be seen in Supplementary Material E. We also plot the absolute variance components in Supplementary Material E. In absolute terms, the total amount of variance is reduced in the difference scores often by a factor of 3 or 4 relative to their components. This is desirable in an experimental task, in which *any* variation in the effect of interest is detrimental.

## How does accounting for reliability affect between-task correlations?

As noted in the introduction, the reliability of two measures will attenuate the magnitude of the correlation that can be observed between them. As an illustration of this phenomenon, we examine the correlations between the four response control tasks administered in Studies 1 and 2 before and after accounting for the reliability of the measures. Response control provides a useful illustrative example of this issue, as it is often assumed that a common response control trait underlies performance on these tasks (for a review, see Bari & Robbins, 2013), though this assumption has received mixed support from correlational research (Aichert et al., 2012; Cyders & Coskunpinar, 2011; Fan, Flombaum, McCandliss, Thomas, & Posner, 2003; Friedman & Miyake, 2004; Hamilton et al., 2015; Ivanov, Newcorn, Morton, & Tricamo, 2011; Khng & Lee, 2014; Scheres et al., 2004; L. Sharma et al., 2014; Stahl et al., 2014; Wager et al., 2005).

Spearman’s Rho correlations can be seen in Table 3. We combined the data from Studies 1 and 2 to maximize statistical power. In order to examine the impact of reliability, in Table 4, we also estimated the dissatenuated correlation coefficients using Spearman’s (1904) formula:

**Table 3:** Spearman’s rho correlations between measures of response control. Data are combined across Study 1 and 2 (total N = 99–104), and averaged across sessions 1 and 2. Correlations significant at p<.05 are highlighted

	Flanker RT cost	Flanker Error cost	Stroop RT cost	Stroop Error cost	Go/no-go Com.
Flanker RT cost					
Flanker Error cost	.29**				
Stroop RT cost	.14	-.14			
Stroop Error cost	-.10	-.01	.28**		
Go/no-go Com.	-.14	.18	-.14	.05	
SSRT Int.	-.14	.14	-.06	-.01	.52***

***p<.001

**p<.01

*p<.05

*RT* reaction time, *Go*/*no*-*go Com*. commission errors in the go/no-go task, *SSRT Int*. stop signal reaction time calculated using the integration method

**Table 4:** Disattenuated Spearman’s rho correlations between measures of response control. Correlations that would be significant at p<.05 (N=100) are highlighted

	Flanker RT cost	Flanker Error cost	Stroop RT cost	Stroop Error cost	Go/no-go Com.
Flanker RT cost					
Flanker Error cost	.50*				
Stroop RT cost	.25*	-.21*			
Stroop Error cost	-.20*	-.02	.51*		
Go/no-go Com.	-.22*	.25*	-.21*	.09	
SSRT Int.	-.31*	.26*	-.11	-.03	.90*

*RT* reaction time, *Go*/*no*-*go Com*. commission errors in the go/no-go task, *SSRT Int*. stop signal reaction time calculated using the integration method

$$ " True" correlation\left( x, y\right)=\frac{Sample\; correlation\left( x, y\right)}{\sqrt{Reliability(x). Reliability(y)}} $$

Spearman noted that the correlation that is observed between two measures will be attenuated (weakened) by measurement error. Assuming that the reliability coefficient reflects the noise in each measure individually, he proposed the disattenuation formula as a means to “correct” the correlation obtained from a sample. As the formula depends on sample estimates of the correlation and reliabilities, it is itself an estimate, and not intended here for inference (for discussions of interpretative issues, see Muchinsky, 1996; Winne & Belfry, 1982). We present them to illustrate the impact of reliability on theoretical conclusions, especially when using the traditional approach of statistical thresholds, though the attenuation of effect sizes is not unique to the null hypothesis significance testing framework. For ease of comparison, correlations significant at p<.05 are highlighted.

Focusing first on the observed correlations in Table 3 there is little support for a relationship between these measures. Consistent with some observations (Reynolds, Ortengren, Richards, & de Wit, 2006), though inconsistent with others (Aichert et al., 2012), we observed a strong correlation between SSRT and commission errors on the go/no-go task. Otherwise, if we were making a dichotomous decision as to whether different response control tasks were related, we would fail to reject the null hypothesis by traditional standards.

The disattenuated correlations in Table 4 paint a somewhat different picture. Note that the dissatenuated correlation will *always* be higher than the observed correlations when the reliabilities are less than one. The increase in the correlations in Table 4 is therefore unsurprising. If we apply the same statistical thresholds however, the dissatenuated correlations lead us to different qualitative conclusions about the relationships between measures. Note that not all of these relationships are consistent with a single underlying response control construct. For example, whereas SSRT shows a positive correlation with flanker error costs, it shows a negative correlation with flanker RT costs. These may suggest other factors moderating the relationships between these measures, such as speed-accuracy trade-offs that carry some consistency across tasks.

For reference, we include the raw and disattenuated correlations for the measures used in Study 3 in the Supplementary Material F.

## Discussion

Across many research, educational, or clinical contexts, when finding a group level effect, it is often theoretically meaningful to ask what factors of the individual predict effectiveness. It is not intuitive, and rarely discussed, that such questions may be at odds with each other because one requires low and one requires high variability between individuals (Rogosa, 1988), even though the statistical issues have been long known. The challenges highlighted by our data are also cause to reflect upon the way in which researchers evaluate paradigms for this purpose; it should not be assumed that robust experimental paradigms will translate well to correlational studies. In fact, they are likely to be sub-optimal for correlational studies for *the same reasons* that they produce robust experimental effects. Our findings, as well as observations from elsewhere in the literature, indicate that this challenge currently exists across most domains of cognitive neuroscience and psychology (De Schryver, Hughes, Rosseel, & De Houwer, 2016; Hahn et al., 2011; Lebel & Paunonen, 2011; Ross et al., 2015). We discuss the practical and theoretical implications of this below, including the way in which sub-optimal reliabilities should be interpreted; the extent to which these problems generalize to other populations; and the challenge this poses to resource intensive research such as neuroimaging, where it is not easy just to increase participant numbers.

## Translating experimental effects to correlational studies

The reliability of a measure is an empirical question and a prerequisite for effective correlational research. Clearly reliability cannot be assumed on the basis of robustness in within-subject contexts. Success in within-subject contexts does not *necessarily* exclude a task from consideration in individual differences contexts, or vice versa. Hypothetically, an effect could produce reliable between-subject variation, but also a mean difference large enough so that it can be consistently reproduced across different samples. However, the reliabilities of many the measures reported here, spanning the domains of attention, cognitive control, and processing style, are much lower than most researchers would expect, and fall short of outlined standards (Barch et al., 2008; Cicchetti & Sparrow, 1981; Fleiss, 1981; Landis & Koch, 1977). There are direct implications of this for initiatives recommending and employing some of the measures we evaluated (e.g., the Stroop and stop-signal tasks; Barch, Braver, Carter, Poldrack, & Robbins, 2009; Hamilton et al., 2015), and for the way in which experimental tasks are evaluated for this purpose in the future.

It is important to emphasize that these results do not indicate that these paradigms are not *replicable*, *valid*, *or robust* measures of their respective constructs. For example, the global precedence effect from the Navon task was highly robust, and generally of a similar magnitude in each session of each study. It also does not preclude the use of these tasks for examining between-group differences in experimental designs. The difference between group means may be sufficiently large so as to be detectable, for example, if one or both groups are located at extreme points on the continuum. Rather, our results suggest that these measures do not consistently distinguish between individuals within a population. Such difficulties with inter-task correlations and reliability have been discussed previously in studies of executive functioning, in the context of the “task impurity” problem (Friedman & Miyake, 2004; Miyake et al., 2000). Individual differences in a given task will likely capture only a subset of “executive functions,” in addition to domain specific mechanisms. Moreover, as Cronbach (1957) highlighted, the goal of the experimentalist is to minimize individual differences, and many of the tasks we examine come originally from this tradition. As a result, these tasks may tap in to aspects of executive functioning that are relatively consistent across individuals compared to those that differentiate between them.

In noting that measures are constructed to achieve different aims in experimental and correlational research, we can also consider whether it is problematic to attempt to experimentally manipulate behavior on measures constructed to reliably measure individual differences. For example, self-report measures such as the UPPS-P are developed with the explicit purpose of assessing stable traits (Whiteside & Lynam, 2001), such that they should be purposefully robust to natural or induced situational variation. Nevertheless, some studies have looked at the UPPS-P dimensions as outcome variables, for example, in a longitudinal study on alcohol use (Kaizer, Bonsu, Charnigo, Milich, & Lynam, 2016). As noted previously, whether a measure is effective for a given aim is an empirical question, though we believe these broader considerations can provide useful guidance.

## Difficulties with difference scores

Statistical concerns regarding the reliability of difference scores in correlational research have been noted previously (Caruso, 2004; Cronbach & Furby, 1970; Lord, 1956). Generally speaking, the difference between two measures is less reliable than the individual measures themselves when the measures are highly correlated and have similar variance (Edwards, 2001; Rogosa, 1988, 1995; Willet, 1988; Zimmerman & Williams, 1998; Zumbo, 1999). In part, this reflects the propagation of error from two component measures to the composite score, but the main reason is that any subtraction that successfully reduces between-participant variance (and thus reduces “error,” as defined in experimental research) is likely to increase the proportion of measurement error relative to between-participant variance (see Fig. 4). In within-subject designs, we often subtract a baseline of behavioral performance or neural activity precisely because we *expect* strong correlations between participants’ performance in multiple conditions, and thus by definition the subtraction will reduce between participant variance relative to error variance. There are notable exceptions in our data with the Flanker and Navon task error scores. Errors in congruent trials in these tasks are uncommon, and there is little variation in the baseline. As such, the difference score primarily reflects incongruent errors. The same is not true of RTs, where individuals strongly co-vary in their responses to congruent and incongruent trials.

However, it does not follow that tasks without difference scores are preferable. In principle, subtracting a baseline measure in order to control for *unwanted* between-participant variance is not at odds with the goal of examining individual differences in performance on that task. After all, one wants to measure individual differences in a specific factor, not just obtain any between-participant variance. For example, simple and choice RTs correlate with measures of general intelligence (Deary, Der, & Ford, 2001; Jensen, 1998). Omitting the baseline subtraction from a task could produce between-task correlations for this reason, but would not aid our understanding of the specific underlying mechanism(s).

## The impact of reliability on statistical power – is “good” good enough?

The past decade has seen increasing attention paid to the failure of the biomedical sciences to always appropriately consider statistical power (Button et al., 2013b; Ioannidis, 2005). Reliability is a crucial consideration for power in correlational research, and the importance of reliable measurement has been emphasized in many landmark psychometric texts (e.g., Guilford, 1954; Gulliksen, 1950; Nunnally, 1970). Despite this, there are no definitive guidelines for interpreting reliability values (Crocker & Algina, 1986). While .6 is nominally considered good by commonly cited criteria (Cicchetti & Sparrow, 1981; Fleiss, 1981; Landis & Koch, 1977), more conservative criteria have been given as a requirement for the use of cognitive tasks in treatment development, citing a minimum of .7 and optimal value of .9 (Barch et al., 2008). Nevertheless, it has been argued that the issue of reliability has been somewhat trivialised in contemporary personality research, with one review noting that “…researchers almost invariably concluded that their stability correlations were ‘adequate’ or ‘satisfactory,’ regardless of the size of the coefficient or the length of the retest interval.” (Watson, 2004, p.326). Researchers might also assume that RT-based measures are inherently more noisy than self-report (e.g., Lane, Banaji, Nosek, & Greenwald, 2007), and that holding all measures to a clinical standard is overly restrictive (Nunnally, 1978). While there may be some truth to these positions, it does not preclude consideration of the implications of poor reliability.

An immediate consequence of a failure to consider reliability in correlational studies is that effect sizes will generally be underestimated. If a researcher conducts an a priori power analysis without factoring in reliability, they bias themselves towards finding a null effect. A less intuitive consequence is that the published literature can *overestimate* effects (Loken & Gelman, 2017). Though on average correlation estimates are attenuated by measurement error, noise can also produce spuriously high correlations on occasion. When spuriously high estimates are selected for by a bias to publish significant findings the average published correlation becomes an overestimate. In combination, these factors are challenges to both reproducibility and theoretical advancement.

Consideration of reliability is not completely absent from the cognitive and imaging literature (e.g., Salthouse, McGuthry, & Hambrick, 1999; Shah, Cramer, Ferguson, Birn, & Anderson, 2016; Yarkoni & Braver, 2010). However, our informal discussions with colleagues and peers suggest that it is not routine to factor reliability estimates into power analyses, and it is exceedingly rare to see this reported explicitly in published power calculations. It is also potentially problematic that researchers tend to underestimate the sample sizes necessary to detect small effects (Bakker, Hartgerink, Wicherts, & van der Maas, 2016). To illustrate these issues concretely, Table 5 shows some numerical examples of the impact of different reliabilities on sample size calculations. This compares the sample size required for the assumed underlying correlation with that required for the attenuated correlation. This calculation, sometimes attributed to Nunnally (1970), rearranges Spearman’s (1904) correction for attenuation formula that we applied earlier:

**Table 5:** The relationship between the true correlation, reliabilities, and observable correlation in two variables. The “True r” is the correlation we would expect to observe given a reliability of 1 for both measures. The “N true” is the sample size that would be required to observe the underlying effect, which is what is normally reported from power calculations. The “Observable r” is the expected correlation after accounting for reliability, corresponding to a recalculated sample size requirement (N obs.). Power calculations were performed using G*Power (Faul, Erdfelder, Buchner, & Lang, 2009; Faul, Erdfelder, Lang, & Buchner, 2007), assuming α = .05 and β = .8

	Reliability				
True *r*	Measure A	Measure B	Observable *r*	N true	N obs.
.7	.8	.8	.56	13	22
.7	.6	.6	.42	13	42
.7	.4	.7	.37	13	55
.5	.8	.8	.4	29	46
.5	.6	.6	.3	29	84
.5	.4	.7	.26	29	113
.3	.8	.8	.24	84	133
.3	.6	.6	.18	84	239
.3	.4	.7	.16	84	304

$$ r\left( measure\kern0.5em  A,\kern0.5em  measure\kern0.5em  B\right)= r\left( true\kern0.5em  A, true\kern0.5em  B\right)\sqrt{reliability\kern0.5em \left( Measure\kern0.5em  A\right) reliability\left( Measure\kern0.5em  B\right)} $$

Two things are apparent from Table 5. First, the magnitude of reliability for a measure has a substantial impact on required sample sizes. Even for reliability nominally considered to be “good” (>.6) by commonly cited criteria (Cicchetti & Sparrow, 1981; Fleiss, 1981; Landis & Koch, 1977), the required sample sizes are about three times higher than what would be specified if reliability had not been taken in to account. Second, even with moderate (*r* = .3) true effect sizes assumed, the sample sizes required greatly exceed those typically used in most cognitive and neurophysiological research.

## Challenges for cognitive neuroscience and clinical research

Though the required sample sizes indicated in Table 5 are not insurmountable in all research contexts, they are particularly challenging for areas that are resource intensive, or access to participants is difficult. Concerns about measurement reliability has also been raised in neuroimaging (e.g., Bennett & Miller, 2010; Mikkelsen, Singh, Sumner, & Evans, 2015; Vul, Harris, Wimkielman, & Pashler, 2009; Wang, Abdi, Bakhadirov, Diaz-Arrastia, & Devous, 2012). For example, it has been estimated that the average reliability of voxel-wise blood-oxygen-level-dependent functional magnetic resonance imaging is .5 (Bennett & Miller, 2010). This is similar to the average of the estimates for our behavioral measures (.45). Assuming reliabilities of .5 for both measures and a large (R= .5) “true” underlying correlation, a sample size of 123 would be required to adequately power correlations between cognition and functional imaging. Such sample sizes are rare, including in our own previous work (Boy, Evans, et al., 2010; see also Yarkoni and Braver, 2010).

Given the prohibitive time and costs of behavioral, imaging, and neuropsychological studies, one might question the utility of pursuing individual differences research. It has been argued that it is not optimal to pursue large sample sizes in neuroimaging because effects that require large samples are not sufficiently large to be of practical or theoretical importance (Friston, 2012, though see commentaries; Button et al., 2013a; Friston, 2013; Ingre, 2013; Lindquist, Caffo, & Crainiceanu, 2013). The extent to which an effect size is considered meaningful will vary according to the research question, though there is little guidance on what our normative expectations should be. A recent meta-analysis of 708 correlations in personality and behavioral research observed that <3% of effects were large by Cohen’s (1988) commonly cited criteria of .5, and 75% of effects were .29 and below (Gignac & Szodorai, 2016). There is certainly a higher range of effect sizes reported in imaging studies (e.g., Vul et al., 2009), though it is likely that these are inflated by the prevalence of small samples, publication bias and questionable research practices (Button et al., 2013b; John, Loewenstein, & Prelec, 2012). Therefore, we believe that the effect sizes and sample sizes reported in Table 5 are representative, even optimistic, for the ranges common to most research questions.

## Measurement error or state-dependence

We have largely discussed issues of task construction and measurement. An alternative possibility is that participants simply fluctuate in their ability to perform these tasks over time and contexts. There is evidence, for example, that SSRTs are sensitive to strategic changes (Leotti & Wager, 2010), and that SSRTs and go/no-go performance are disrupted by alcohol (e.g., Caswell, Morgan, & Duka, 2013; de Wit, Crean, & Richards, 2000; Dougherty, Marsh-Richard, Hatzis, Nouvion, & Mathias, 2008; Mulvihill, Skilling, & VogelSprott, 1997; Weafer & Fillmore, 2008), indicating that performance on these tasks is not impermeable.

Nevertheless, there is evidence for stability for some tasks in our data. Low ICCs in a homogenous sample are not necessarily indicative of substantial *changes* in performance. The low SEMs in the flanker RT cost indicate that participants generally perform the task similarly in both sessions, even though the relative ranking between individuals is not consistent. Further, if the low ICCs we observe were primarily due to variation in psychological or physiological factors over the course of 3 weeks, we might expect high reliabilities when comparing performance in the first half of each session to the second half, or comparing odd and even numbered trials. However, these within-session reliabilities (Supplementary Material G) show similarly sub-optimal reliability for the key measures (see also Khng & Lee, 2014). An exception to this is the stop-signal reaction time, where the odd vs. even trial comparison produces estimates between .82 and .89 for the integration method. This is likely in part because the tracking procedure used will produce a high reliability for the SSD when taking alternating trials.

We would generally expect measurements taken closely together in time to yield higher estimates of reliability than those taken at more distant points, even within a single testing session. However, there are sources of variance outside the construct of interest that could increase or decrease reliability estimates. Time-series analysis of RTs suggests that there is a correlation between the speeds of consecutive responses given by an individual, which decreases as the number of intervening trials increases (Gilden, Thornton, & Mallon, 1995; Wagenmakers, Farrell, & Ratcliff, 2004). Estimates comparing odd to even numbered trials may appear to be more reliable because they encompass such short-scale fluctuations. Alternatively, factors such as practice effects or fatigue may decrease reliability by increasing measurement error, or by producing systematic shifts in performance between measurement points (e.g., individuals being slower in the second half of trials compared to the first). The analyses we conduct in Supplementary Materials D and G explore these as possible reasons for the sub-optimal reliabilities that we observed. Taken together, these suggest that the key issue is simply that individuals do not differ enough from one another to reliably overcome measurement fluctuations.

## Generalizability of findings to other populations.

If between-participant variance differs markedly between populations, the population with higher variance will show higher reliability, unless measurement noise increases proportionally. We used a (predominantly female) student sample, who might show restricted variance compared to a general population. However, our comparisons indicate that they have similar levels of variability to samples taken from a general population, which also did not show consistently higher reliability estimates (see Supplementary Material C1 and C2). Further, the components of UPPS-P, a self-report measure of impulsivity, showed reliabilities between .7–.9, indicating that reliable measurement is attainable in a student sample on measures designed to differentiate between individuals. Finally, examples of sub-optimal reliability for robust within-subject effects are not limited to student samples (e.g., attention networks in schizophrenic patients and healthy controls; Hahn et al., 2011). Therefore, the issues we discuss are likely to generalize to other samples.

Though our sample sizes are larger than many previous retest reliability studies of these tasks, it has been argued that samples approaching 250 are necessary for a stable estimate of the (Pearson’s) correlation effect size (Schonbrodt & Perugini, 2013). Using simulations, they defined stability as the point at which the “observed” correlation did not deviate from a specified window (±.1) around the “true” effect with the addition of more data points. However, the point of stability is dependent on the size of the underlying correlation, and the degree of uncertainty one is willing to accept. For example, assuming a confidence (power) level of 80% and a population correlation of R = .7, the point of stability for a window of ±.15 was N=28. Therefore, ICCs as low as the ones we observe are unlikely if the population ICC is excellent.

The student population we used is typical of most cognitive and imaging studies, but regardless of population, the main points of this paper will remain true: experimental designs aim to minimize between-subject variance, and thus successful tasks in that context should be expected to have low reliability; taking reliability into account could entirely change theoretical inferences from correlational structure.

## Future directions and recommendations

Our consideration of reliability issues form part of a broader concern that studying individual differences is challenging for laboratory-based research, particularly in resource-intensive contexts such as neuroimaging. With these global issues in mind, we discuss approaches that could help to optimize research designs using cognitive tasks. Note that although the majority of discussion focuses on analysis methods, one should not expect to create inter-subject variability from a task that is designed to produce homogenous performance. Researchers should be mindful of these properties at the stages of task design/selection and power analysis. For several of these approaches, it is undetermined or untested whether they improve reliability estimates for the contexts we focus on here, though some have shown promise in other areas.

### Alternative measurement approaches

The independent examination of mean RTs or mean error rates belies the richness of the data provided by many behavioral tasks. The practice of considering RT and errors costs as independent and interchangeable measures of performance has been questioned in several areas (e.g., Draheim, Hicks, & Engle, 2016; Ratcliff & Rouder, 1998; Wickelgren, 1977). In the domain of task switching, it has been suggested that composite scores of RT costs and error rates are better able to predict performance in a working memory task than RT costs alone (Draheim et al., 2016; Hughes, Linck, Bowles, Koeth, & Bunting, 2014). Further, Hughes et al. observed higher within-session reliabilities for composite RT-accuracy scores, relative to RT costs or accuracy costs in isolation, but only when using a response deadline procedure.

Alternatively, mathematical models of decision making such as the drift-diffusion model (Ratcliff, 1978; Ratcliff & Rouder, 1998; Ratcliff, Smith, Brown, & McKoon, 2016) decompose RT and accuracy into parameters thought to reflect decision processes. The combination of modelling techniques with imaging methods has also been discussed (Forstmann, Ratcliff, & Wagenmakers, 2016; Forstmann & Wagenmakers, 2015). Recently, Lerche and Voss (2017) observed that the retest reliability of key diffusion model parameters was similar to that of overall accuracy and mean RT in lexical decision, recognition memory, and an associative priming task. However, the parameters they extracted reflect processes (e.g., information processing speed) in individual conditions or across conditions, rather than a within-subject effect analogous to an RT cost. It is possible to create difference scores from model parameters, though these may be subject to the same statistical issues noted previously. Thus, while there may be theoretical value in such modelling approaches, whether they improve reliability estimates for experimental effects is an open question.

Another suggested alternative to difference scores is to use residualized differences (Cronbach & Furby, 1970; DuBois, 1957; Friedman & Miyake, 2004). This entails a regression approach in which scores in the baseline condition (e.g., congruent RT) are used to predict incongruent RTs, and an individual’s residual from their predicted value is taken as the index of performance. Residualized scores show improved reliability over standard difference scores in *some* situations, though their interpretation is not straightforward (for a review, see Willet, 1988). Evaluating the theoretical strengths and weaknesses of all these approaches is beyond the scope of the current paper. From a methodological perspective, the reliability of any composite measure or modelled parameter will not be perfect, and thus needs to be empirically measured and accounted for.

### Alternative statistical approaches

In our reliability analyses, we adopted the ANOVA-based approach to estimating components of variance (McGraw & Wong, 1996; Shrout & Fleiss, 1979). This is perhaps the most commonly used method in psychology, produced by popular packages such as SPSS. Variance components can alternatively be estimated via the use of linear mixed-effects (LMMs) and generalized linear mixed-effects models (GLLMs; Nakagawa & Schielzeth, 2010). These models allow greater flexibility in dealing with distributional assumptions and confounding variables. Structural equation models have also grown increasingly popular in psychology (Anderson & Gerbing, 1988) as a method to examine relationships between constructs theorized to underlie observable behaviors (Anderson & Gerbing, 1988). Factor analysis and structural equation modelling have been used previously to examine commonality among response inhibition and executive functioning tasks (see, e.g., Aichert et al., 2012; Friedman & Miyake, 2004; Stahl et al., 2014). An attractive feature of this approach is they allow for measurement error to be modelled separately from variance shared between measures. Latent variable models have also been applied to reliability estimates in the form of latent state-trait models. (Newsom, 2015; Steyer, Schmitt, & Eid, 1999; Steyer & Schmitt, 1990). They typically use data from three or more sessions, and can dissociate variance that is stable across sessions from session specific and residual (error) variance. Notably, one study has also applied this approach to the parameters of the drift-diffusion model derived from multiple tasks (Schubert, Frischkorn, Haemann, & Voss, 2016). A limiting factor is that structural equation models typically require large samples, with suggestions typically falling in the 100s (c.f. Wolf, Harrington, Clark, & Miller, 2013). This, in addition to the time required to administer multiple tasks or sessions, may make the approach infeasible for many researchers. Finally, Item Response Theory (IRT; see, e.g., Hambleton, Swaminathan, & Rogers, 1991; Lord & Novick, 1968) has arguably superseded classical test theory in educational testing. The goal of IRT is to characterize the relationship between typically a single latent trait (e.g., maths ability) and the probability of a binary response (e.g., correct or incorrect) on individual test items. The resulting item response curve captures both the location of each item with respect to the latent trait (i.e., its difficulty), and the sensitivity of the item to differing levels of ability (i.e., its slope). Though not easily applicable to the current format of most experimental tasks, the contribution of IRT to educational testing is notable if constructing new tests for the purposes of cognitive and clinical measurement.

### Interactions in experimental designs

In addition to factoring reliability into power calculations as detailed above, within-subject designs can be used to examine associations and dissociations between measures. For example, the absence of correlations in our data between SSRT and the Stroop task implies no relationship between performance in these tasks. In contrast, shared mechanisms have been implicated in experimental studies that have combined the tasks, where Stroop stimuli are used in place of the typical two choice stimuli used in the SST (Kalanthroff, Goldfarb, & Henik, 2013; Verbruggen, Liefooghe, & Vandierendonck, 2004). Verbruggen et al. observed longer SSRTs on incongruent trials relative to neutral trials, suggesting that the mechanisms underlying the resolution of conflict between stimuli overlaps with the mechanisms underlying response inhibition in the SST. Within-subject designs may be more appropriate to examine interactions and dissociations between underlying mechanisms when individual differences per se are not the primary focus (for further examples in cognitive control and other areas, see, e.g., Awh, Vogel, & Oh, 2006; Boy, Husain, et al., 2010; Hedge, Oberauer, & Leonards, 2015).

## Conclusions

In concluding their prominent discussion of the reliability of difference scores, Cronbach and Furby (1970) offered the advice, “It appears that investigators who ask questions regarding gain scores would ordinarily be better advised to frame their questions in other ways” (p. 80). This damning statement has been qualified in subsequent work (Rogosa, 1988; Zimmerman & Williams, 1998; Zumbo, 1999), though as illustrated by our findings, robust experimental effects do not necessarily translate to optimal methods of studying individual differences. We suggest that this is because experimental designs have been developed and naturally selected for providing robust effects, which means low between-participant variance. Cronbach (1957) called for a bridging of the gap between experimental and correlational research in psychology, and we support this goal. However, our findings suggest more caution is required when translating tools used to understand mechanisms in one context to the other.

## Electronic supplementary material

Below is the link to the electronic supplementary material.

ESM 1 (DOCX 4.96 mb)

## Footnotes

1 The two-way ICC can be calculated for absolute agreement or for consistency of agreement. The latter omits the between-session variance term. Note also that the error variance term does not distinguish between measurement error and non-systematic changes in the individuals’ true scores (Heize, 1969). Some may therefore prefer to think of the coefficient as an indicator of stability.
