# Supplementary material for: The reliability paradox: Why robust cognitive tasks do not produce reliable individual differences
Source: Behav Res Methods. 2017 Jul 19;50(3):1166–86. doi: 10.3758/s13428-017-0935-1 (PMC5990556; doi:10.3758/s13428-017-0935-1)
Supplement: Supplementary file 1 — (DOCX 4.96 mb) [file 13428_2017_935_MOESM1_ESM.docx]

**Supplementary material A**

**Alternative reliability calculations**

This table contains reliability estimates for our measures calculated with different methods. The ICC agreement in the first column is as reported in text, though we combine Studies 1 and 2 here. This ICC is calculated using the ANOVA method, and is the ratio of between-participant variance to the total variance (between-participant, between-session, and error variance). The ICC for consistency omits the between-session variance from the denominator, therefore, it reflects whether participants maintain their order and spacing irrespective of mean differences between sessions (e.g. the sample being faster due to practice effects). For the jackknife estimate, we repeated the ICC for agreement calculation N-1 times with each participant excluded, with N being the total sample size. We give the mean, minimum and maximum of these estimates. This gives an indication of whether our estimates are affected by outliers. Rho values are the Spearman’s Rho correlation estimates. Finally, the medians are the ICC for agreements calculated using medians rather than means for the RT measures. In general, all of these approaches give similar reliability estimates.

Note, we do not combine the UPPS-P findings from Studies 1 and 2 with Study 3, because participants from the first two studies were not excluded from taking part in Study 3.

In addition, we also estimated the reliability of stop-signal reaction times using a more conservative cut-off criteria. If any participant whose probability of successfully inhibiting a response to signal trials was significantly different from 50% (remaining N=79), the ICCs for agreement were .40 for both go RTs and SSRTi.

Supplementary Table A1. Retest reliability for studies 1 and 2 combined, and study 3. See text above for details.

|  |  |  |  | Jackknife estimate | | |  |  |
| --- | --- | --- | --- | --- | --- | --- | --- | --- |
|  |  | ICC agreement | ICC consistency | Mean | Min | Max | Rho | Medians |
| Flanker | Congruent RT | 0.72 (0.5-0.83) | 0.76 (0.67-0.83) | 0.71 | 0.68 | 0.74 | 0.71 | 0.72 (0.51-0.83) |
|  | Neutral RT | 0.68 (0.43-0.81) | 0.74 (0.63-0.81) | 0.68 | 0.63 | 0.7 | 0.68 | 0.70 (0.46-0.82) |
|  | Incongruent RT | 0.64 (0.36-0.78) | 0.70 (0.59-0.79) | 0.64 | 0.6 | 0.66 | 0.67 | 0.65 (0.39-0.79) |
|  | **RT cost** | **0.50 (0.31-0.64)** | **0.53 (0.38-0.66)** | **0.50** | **0.48** | **0.55** | **0.62** | **0.48 (0.29-0.62)** |
|  | Congruent errors | 0.41 (0.23-0.56) | 0.42 (0.25-0.57) | 0.41 | 0.34 | 0.46 | 0.65 |  |
|  | Neutral errors | 0.41 (0.23-0.56) | 0.44 (0.27-0.58) | 0.41 | 0.33 | 0.46 | 0.64 |  |
|  | Incongruent errors | 0.63 (0.47-0.74) | 0.65 (0.53-0.75) | 0.63 | 0.60 | 0.67 | 0.71 |  |
|  | **Error cost** | **0.66 (0.53-0.75)** | **0.66 (0.53-0.76)** | **0.66** | **0.64** | **0.68** | **0.63** |  |
| Stroop | Congruent RT | 0.74 (0.52-0.85) | 0.79 (0.71-0.85) | 0.74 | 0.73 | 0.77 | 0.79 | 0.74 (0.57-0.84) |
|  | Neutral RT | 0.73 (0.43-0.86) | 0.8 (0.72-0.86) | 0.73 | 0.71 | 0.76 | 0.81 | 0.74 (0.49-0.85) |
|  | Incongruent RT | 0.69 (0.2-0.86) | 0.8 (0.71-0.86) | 0.69 | 0.67 | 0.71 | 0.79 | 0.69 (0.29-0.84) |
|  | **RT cost** | **0.64 (0.32-0.8)** | **0.72 (0.61-0.8)** | **0.64** | **0.61** | **0.66** | **0.7** | **0.50 (0.24-0.68)** |
|  | Congruent errors | 0.39 (0.21-0.55) | 0.42 (0.25-0.57) | 0.39 | 0.36 | 0.45 | 0.46 |  |
|  | Neutral errors | 0.48 (0.29-0.62) | 0.51 (0.35-0.64) | 0.48 | 0.45 | 0.53 | 0.57 |  |
|  | Incongruent errors | 0.51 (0.34-0.64) | 0.52 (0.36-0.65) | 0.51 | 0.47 | 0.54 | 0.56 |  |
|  | **Error cost** | **0.46 (0.29-0.6)** | **0.46 (0.29-0.6)** | **0.46** | **0.43** | **0.50** | **0.44** |  |
| Go/No-go | Go RT | 0.67 (0.56-0.77) | 0.68 (0.56-0.77) | 0.67 | 0.65 | 0.8 | 0.76 | 0.58 (0.44-0.70) |
|  | **Commission errors** | **0.76 (0.63-0.84)** | **0.78 (0.7-0.85)** | **0.76** | **0.73** | **0.79** | **0.78** |  |
|  | Omission errors | 0.48 (0.32-0.62) | 0.49 (0.33-0.62) | 0.48 | 0.36 | 0.60 | 0.43 |  |
| Stop-signal | Go RT | 0.43 (0.22-0.59) | 0.47 (0.3-0.61) | 0.43 | 0.41 | 0.5 | 0.71 |  |
|  | Mean SSD | 0.41 (0.24-0.56) | 0.43 (0.25-0.58) | 0.41 | 0.39 | 0.48 | 0.64 |  |
|  | **SSRT mean** | **0.45 (0.28-0.6)** | **0.47 (0.3-0.61)** | **0.45** | **0.42** | **0.50** | **0.53** |  |
|  | **SSRT integration** | **0.43 (0.26-0.58)** | **0.44 (0.27-0.59)** | **0.43** | **0.36** | **0.48** | **0.53** |  |
| UPPS-P | Negative U. | 0.72 (0.62-0.8) | 0.72 (0.62-0.8) | 0.72 | 0.70 | 0.74 | 0.72 |  |
|  | Premeditation | 0.78 (0.69-0.85) | 0.79 (0.7-0.85) | 0.78 | 0.76 | 0.8 | 0.73 |  |
|  | Perseverance | 0.75 (0.65-0.83) | 0.75 (0.65-0.82) | 0.75 | 0.71 | 0.8 | 0.69 |  |
|  | Sensation Seek. | 0.88 (0.83-0.92) | 0.88 (0.83-0.92) | 0.88 | 0.87 | 0.91 | 0.9 |  |
|  | Positive U. | 0.81 (0.74-0.87) | 0.81 (0.73-0.87) | 0.81 | 0.8 | 0.83 | 0.81 |  |
| Posner | Valid RT | 0.80 (0.61-0.9) | 0.83 (0.7-0.91) | 0.8 | 0.69 | 0.82 | 0.69 | 0.78 (0.56-0.88) |
|  | Invalid RT | 0.79 (0.56-0.89) | 0.82 (0.69-0.9) | 0.79 | 0.74 | 0.81 | 0.74 | 0.78 (0.52-0.89) |
|  | **Cueing effect** | **0.70 (0.5-0.83)** | **0.72 (0.52-0.84)** | **0.7** | **0.56** | **0.78** | **0.64** | **0.73 (0.54-0.85)** |
| SNARC | Congruent RT | 0.69 (0.49-0.82) | 0.69 (0.48-0.82) | 0.69 | 0.62 | 0.78 | 0.79 | 0.67 (0.46-0.81) |
|  | Incongruent RT | 0.74 (0.56-0.86) | 0.76 (0.58-0.86) | 0.74 | 0.69 | 0.78 | 0.67 | 0.72 (0.54-0.84) |
|  | **SNARC effect RT** | **0.22 (-0.08-0.49)** | **0.23 (-0.08-0.5)** | **0.22** | **0.13** | **0.32** | **0.24** | 0.19 (-0.12-0.47) |
|  | Congruent errors | 0.67 (0.45-0.81) | 0.66 (0.45-0.81) | 0.67 | 0.61 | 0.71 | 0.68 |  |
|  | Incongruent errors | 0.58 (0.33-0.75) | 0.57 (0.32-0.75) | 0.58 | 0.5 | 0.64 | 0.5 |  |
|  | **SNARC effect errors** | **0.03 (-0.28-0.34)** | **0.03 (-0.28-0.34)** | **0.03** | **-0.04** | **0.08** | **0.02** |  |
| Navon | Local congruent RT | 0.69 (0.49-0.83) | 0.71 (0.51-0.84) | 0.69 | 0.49 | 0.72 | 0.61 | 0.69 (0.47-0.82) |
|  | Local incongruent RT | 0.68 (0.45-0.83) | 0.72 (0.52-0.84) | 0.68 | 0.62 | 0.7 | 0.69 | 0.71 (0.44-0.85) |
|  | **Local RT cost** | **0.14 (-0.17-0.43)** | **0.14 (-0.18-0.43)** | **0.14** | **0.06** | **0.29** | **0.22** | **0.3 (-0.01-0.55)** |
|  | Local congruent errors | 0.56 (0.3-0.74) | 0.55 (0.29-0.73) | 0.56 | 0.5 | 0.63 | 0.56 |  |
|  | Local incongruent errors | 0.8 (0.65-0.89) | 0.79 (0.64-0.89) | 0.8 | 0.72 | 0.82 | 0.71 |  |
|  | **Local error cost** | **0.82 (0.69-0.9)** | **0.82 (0.68-0.9)** | **0.82** | **0.74** | **0.84** | **0.71** |  |
|  | Global congruent RT | 0.63 (0.4-0.78) | 0.64 (0.41-0.79) | 0.62 | 0.45 | 0.68 | 0.65 | 0.67 (0.46-0.81) |
|  | Global incongruent RT | 0.70 (0.5-0.83) | 0.72 (0.54-0.84) | 0.7 | 0.52 | 0.75 | 0.7 | 0.71 (0.49-9,84) |
|  | **Global RT cost** | **-0.14 (-0.43-0.18)** | **-0.14 (-0.43-0.18)** | **-0.14** | **-0.26** | **-0.06** | **-0.15** | **0.03 (-0.27-0.33)** |
|  | Global congruent errors | 0.6 (0.36-0.76) | 0.61 (0.37-0.77) | 0.6 | 0.55 | 0.66 | 0.59 |  |
|  | Global incongruent errors | 0.71 (0.51-0.84) | 0.73 (0.54-0.85) | 0.71 | 0.69 | 0.74 | 0.73 |  |
|  | **Global error cost** | **0.17 (-0.15-0.46)** | **0.16 (-0.15-0.45)** | **0.17** | **0.03** | **0.24** | **-0.01** |  |
|  | **Global precedence effect (RT)** | **-0.03 (-0.34-0.29)** | **-0.03 (-0.33-0.28)** | **-0.03** | **-0.14** | **0.06** | **0.04** | **0.16 (-0.16-0.45)** |
| UPPS-P | Negative U. | 0.78 (0.63-0.88) | 0.79 (0.63-0.88) | 0.78 | 0.76 | 0.82 | 0.8 |  |
|  | Premeditation | 0.88 (0.78-0.93) | 0.88 (0.78-0.93) | 0.88 | 0.86 | 0.89 | 0.81 |  |
|  | Perseverance | 0.9 (0.81-0.94) | 0.89 (0.81-0.94) | 0.9 | 0.87 | 0.91 | 0.81 |  |
|  | Sensation Seek. | 0.91 (0.83-0.95) | 0.91 (0.83-0.95) | 0.91 | 0.9 | 0.92 | 0.94 |  |
|  | Positive U. | 0.85 (0.67-0.93) | 0.87 (0.78-0.93) | 0.85 | 0.83 | 0.87 | 0.86 |  |

**Supplementary material B**

**Descriptive statistics for Studies 1 -3**

Supplementary Table B1. Means and standard deviations (parentheses) for tasks administered in Studies 1 and 2.

| Task | Measure | Study 1 | | Study 2 | |
| --- | --- | --- | --- | --- | --- |
|  |  | Session 1 | Session 2 | Session 1 | Session 2 |
| Flanker task | Congruent RT | 422 ms (47) | 405 ms (54) | 421 ms (42) | 404 ms (38) |
|  | Neutral RT | 420 ms (46) | 401 ms (53) | 415 ms (41) | 397 ms (34) |
|  | Incongruent RT | 467 ms (55) | 441 ms (64) | 466 ms (55) | 441 ms (47) |
|  | **RT cost** | 46 ms (21) | 35 ms (19) | 44 ms (24) | 37 ms (23) |
|  |  |  |  |  |  |
|  | Congruent % correct | 94.4 % (5.9) | 93.2 % (7) | 95.8 % (4.4) | 92.9 % (8.5) |
|  | Neutral % correct | 93.6 % (6.3) | 92.2 % (7.1) | 95.0 % (4.6) | 91.7 % (8.5) |
|  | Incongruent % correct | 85.2 % (8.3) | 83.9 % (9.1) | 87.3 % (8.3) | 83.5 % (10.3) |
|  | **Error cost** | 9.1 % (5.4) | 9.2 % (6.2) | 8.5 % (5.9) | 9.4 % (6.0) |
| Stroop task | Congruent RT | 611 ms (73) | 582 ms (80) | 609 ms (65) | 586 ms (61) |
|  | Neutral RT | 640 ms (80) | 604 ms (76) | 643 ms (77) | 614 ms (69) |
|  | Incongruent RT | 689 ms (90) | 643 ms (81) | 699 ms (84) | 651 ms (74) |
|  | **RT cost** | 78 ms (38) | 61 ms (34) | 91 ms (51) | 65 ms (44) |
|  |  |  |  |  |  |
|  | Congruent % correct | 93.7 % (3.3) | 92.6 % (5) | 94.6 % (3.6) | 92.6 % (4.6) |
|  | Neutral % correct | 92.7 % (3.5) | 91.3 % (5.8) | 93.1 % (4.3) | 90.9 % (5.2) |
|  | Incongruent % correct | 90.3 % (5.2) | 89.8 % (6.9) | 91.8 % (4.2) | 89.6 % (5.9) |
|  | **Error cost** | 3.4 % (4.3) | 2.7 % (4.4) | 2.9 % (3.1) | 3 % (3.4) |
| Go/No-go task | Go RT | 423 ms (56) | 420 ms (63) | 422 ms (52) | 438 ms (71) |
|  | **Commission errors** | 19.3 % (10.7) | 22.5 % (12.4) | 20.3 % (12.6) | 23.8 % (15) |
|  | Omission errors | 1 % (1.8) | 1.4 % (3.5) | 1.5 % (3.6) | 2.7 % (6) |
| Stop-signal task | Go RT | 517 ms (165) | 457 ms (96) | 494 ms (104) | 448 ms (80) |
|  | Mean SSD | 267 ms (192) | 217 ms (114) | 232 ms (120) | 201 ms (92) |
|  | **SSRT mean** | 259 ms (49) | 251 ms (40) | 272 ms (41) | 260 ms (35) |
|  | **SSRT integration** | 247 ms (51) | 235 ms (47) | 259 ms (51) | 247 ms (47) |
| UPPS-P | Negative U. | 2.38 (0.51) | 2.34 (0.61) | 2.50 (0.56) | 2.42 (0.58) |
|  | Premeditation | 2.05 (0.47) | 2.09 (0.46) | 2 .00 (0.44) | 2.08 (0.51) |
|  | Perseverance | 2.1 (0.54) | 2.04 (0.57) | 1.97 (0.45) | 2.00 (0.45) |
|  | Sensation Seek. | 2.98 (0.67) | 2.96 (0.67) | 2.98 (0.60) | 2.93 (0.70) |
|  | Positive U. | 1.86 (0.52) | 1.88 (0.59) | 2.14 (0.65) | 2.09 (0.69) |

Supplementary Table B2.Means and standard deviations (parentheses) for tasks administered in Studies 3.

| Task | Measure | Session 1 | Session 2 |
| --- | --- | --- | --- |
| Posner task | Congruent RT | 290 ms (36) | 279 ms (40) |
|  | Incongruent RT | 323 ms (53) | 306 ms (50) |
|  | **RT cost** | **33 ms (26)** | **27 ms (22)** |
| SNARC task | Congruent RT | 456 ms (45) | 452 ms (58) |
|  | Incongruent RT | 471 ms (48) | 460 ms (57) |
|  | **RT cost** | **15 ms (20)** | **8 ms (16)** |
|  |  |  |  |
|  | Congruent % correct | 92.8 % (3.8) | 93.2 % (3.2) |
|  | Incongruent % correct | 92.1 % (4.0) | 92 % (4.2) |
|  | **Error cost** | **0.7 % (2.3)** | **1.2 % (2.4)** |
| Navon task | Local congruent RT | 493 ms (59) | 479 ms (50) |
|  | Local incongruent RT | 569 ms (65) | 551 ms (45) |
|  | **Local RT cost** | **76 ms (22)** | **71 ms (18)** |
|  |  |  |  |
|  | Local congruent % correct | 97.6 % (1.8) | 97.6 % (1.9) |
|  | Local incongruent % correct | 84.6 % (9.6) | 84.8 % (9.1) |
|  | **Local error cost** | **13 % (8.9)** | **12.8 % (8.4)** |
|  |  |  |  |
|  | Global congruent RT | 429 ms (63) | 416 ms (49) |
|  | Global incongruent RT | 432 ms (63) | 416 ms (51) |
|  | **Global RT cost** | **3 ms (10)** | **1 ms (15)** |
|  |  |  |  |
|  | Global congruent % correct | 93 % (3.6) | 93.8 % (3.5) |
|  | Global incongruent % correct | 91.7 % (4.3) | 92.7 % (3.2) |
|  | **Global error cost** | **1.3 % (3.0)** | **1.1 % (2.9)** |
|  |  |  |  |
|  | **Global precedence** | **64 ms (22)** | **64 ms (25)** |
| UPPS-P | Negative U. | 2.50 (0.48) | 2.43 (0.48) |
|  | Premeditation | 2.14 (0.39) | 2.16 (0.43) |
|  | Perseverance | 2.18 (0.55) | 2.17 (0.54) |
|  | Sensation Seek. | 3.07 (0.48) | 3.04 (0.56) |
|  | Positive U. | 2.17 (0.58) | 2.01 (0.57) |

**Supplementary material C**

**Comparison of sample characteristics and re-test reliability for response control tasks.**

In this section, we provide comparative data from other studies that have administered response control tasks. Table C1 shows the standard deviation of our student sample compared to samples from the general population reported in the literature. Note that other studies using these tasks (e.g. Stahl et al., 2014) state that their sample was also made up exclusively or predominantly of students. Our sample are not consistently more or less variable than these comparison samples.

Table C2 summarises retest reliability and study information from previous reliability studies on response control tasks. Again, the reliability values we report are not consistently higher or lower than those reported previously. Note that the exact form of the reliability coefficient may vary across studies.

Table C1. Comparison of sample variability on common response control tasks (standard deviations) in our student sample (combined across studies 1 and 2) with samples from the general population reported in the literature.

|  | Session 1 (N=99-104) | Session 2 (N=99-104) | Wöstmann et al. session 1 (N=23) |  | Wöstman et al. session 2 (N=23) | Aichert et al. (2012; N=504) | Penadés et al. (2007; N=25) | Weafer et al. (2013) session 1 (N=121) | Weafer et al. (2013) session 2 (N=121) |
| --- | --- | --- | --- | --- | --- | --- | --- | --- | --- |
| Flanker RT cost | 23 ms | 21 ms | 32 ms |  | 25 ms |  |  |  |  |
| Flanker Error Cost | 6 %* | 6 %** | 2 %* |  | 4 %** |  |  |  |  |
| GNG commission errors | 12 % | 14 % | 15 % |  | 15 % | 14% | 11% |  |  |
| SSRT | 52 ms^†^ | 48 ms^††^ | 84 ms^†^ |  | 122 ms^†^ | 97ms | 98ms | 44ms | 38ms |

* Levene’s test indicates that variance is significantly greater in our sample compared to Wöstmann et al.; W=12.17, p<.001. ** Variance significantly greater in our sample compared to Wöstmann et al.; W=13.46, p<.001. ^†^ Variance significantly greater in Wöstmann et al. sample compared to our sample; W=9.466, p=.003. ^††^ Variance significantly greater in Wöstmann et al. sample compared to our sample; W=24.263 p<.001.

Table C2. Summary of test-retest reliability and study information from literature

| Task | Measure | Study | Reliability | Participant N (age) | Trial number |
| --- | --- | --- | --- | --- | --- |
| Eriksen flanker | RT cost | van Leeuwen et al. (2007) 1 | 0.48 | 74 (8-11 ) | 40 per condition |
|  |  | van Leeuwen et al. (2007) 2 | 0.48 | 28 (4-20 ) | 40 per condition |
|  |  | Wöstmann et al. (2013) | 0.91 | 23 (18-55) | 40 per condition |
|  | Error cost | van Leeuwen et al. (2007) 1 | 0.29 | 74 (8-11 ) | 40 per condition |
|  |  | van Leeuwen et al. (2007) 2 | 0.14 | 28 (14-20 ) | 40 per condition |
|  |  | Wöstmann et al. (2013) | 0.65 | 23 (18-55) | 40 per condition |
|  |  |  |  |  |  |
| Go/no-go | Commission errors | Weafer, Baggott & de Wit (2013) | 0.65 | 123 (18-30) | Not reported |
|  |  | Wöstmann et al. (2013) | 0.84 | 23 (18-55) | 220 go, 80 no-go |
|  |  | Langenecker et al. (2007)* | 0.63 | 28 (mean: 18.9) | Not reported |
|  |  |  |  |  |  |
| Stroop | Interference score (RT)** | Wöstmann et al. (2013) | 0.82 | 23 (18-55) | Three lists |
|  | Interference score (% correct) ** | Kindlon et al. (1995) | 0.67 | 136 (6-16) | Three lists |
|  | RT cost (verbal response) | Strauss et al (2005) | 0.46 | 28 (18-40) | 50 per condition |
|  |  |  |  |  |  |
| Stop-signal task | SSRT | Wöstmann et al. (2013) | 0.03 | 23 (18-55) | 130 go, 48 stop |
|  |  | Kuntsi et al. (2001) | 0.11 | 34 (7.9 - 15.3) | 192 go, 64 stop |
|  |  | Weafer, Baggott & de Wit (2013) | 0.65 | 121 (18-30) | Not reported |
|  |  | Soreni et al. (2009) | 0.72 | 12 (9-15) | 96 go, 32 stop |
|  |  |  |  |  |  |
|  | Prob. Inhibition | Kindlon et al. (1995) | 0.79 | 136 (6-16) | Not reported |
|  | Slope of inhibition function | Kindlon et al. (1995) | 0.72 | 136 (6-16) | Not reported |
|  | Commission errors | Kindlon et al. (1995) | 0.61 | 136 (6-16) | Not reported |
| *Note.* * Reliability for their level 2 reported here. Task variant additionally manipulated set shifting, such that the stimulus that participants withheld a response to varied within a block. **Scores were derived from differences between performances on lists of items, rather than trial by trial. | | | | | |

**Supplementary material D – relationship between reliability and trial number**

A key factor contributing to measurement error, and thus reliability and cross-task correlation, is variability between trials: the stability of the mean estimate for an individual will grow with the number of trials employed, but trial numbers can vary greatly between tasks and studies, are rarely emphasised, and sometimes not even reported. We use our data to evaluate the number of trials at which the reliability estimates stabilise. We adopted a similar subsampling approach to that Schonbrodt and Perugini (2013) used to estimate the impact of participant numbers. First, we plot how retest reliability changes as the number of trials increases *in the order completed by participants*, indicated by the black solid lines in figure 3. The advantage of this approach is that it keeps any practice and fatigue effects as they are, but it remains subject to the noise inherent in trial variability. Then, we estimate reliabilities by sampling randomly from the entire pool of trials (with replacement), doing this 1000 times for each potential number of trials. This approach gives a smooth curve (red line in Figure 3) and 95^th^ percentiles (green lines), but ignores practice and fatigue effects. In order to provide a simple guideline for the number of trials needed for stable reliability, we identified the number of trials at which reliability estimates fell consistently within .1 of the reliability estimated from the entire dataset (black dashed line). Note that most categorical interpretations of ICCs span a range of .2 (e.g. .6 to .8; Cicchetti & Sparrow, 1981; Fleiss, 1981; Landis & Koch, 1977). This is a simplified version of the approach used by Schonbrodt and Preugini (2013). For reference, we indicate the number of trials used in previous reliability studies (orange dashed lines, see Supplementary material A; averaged where n>1), which also correspond to the number used in prominent individual differences studies (e.g., 40 trials per condition in the flanker task; Friedman & Miyake, 2004).


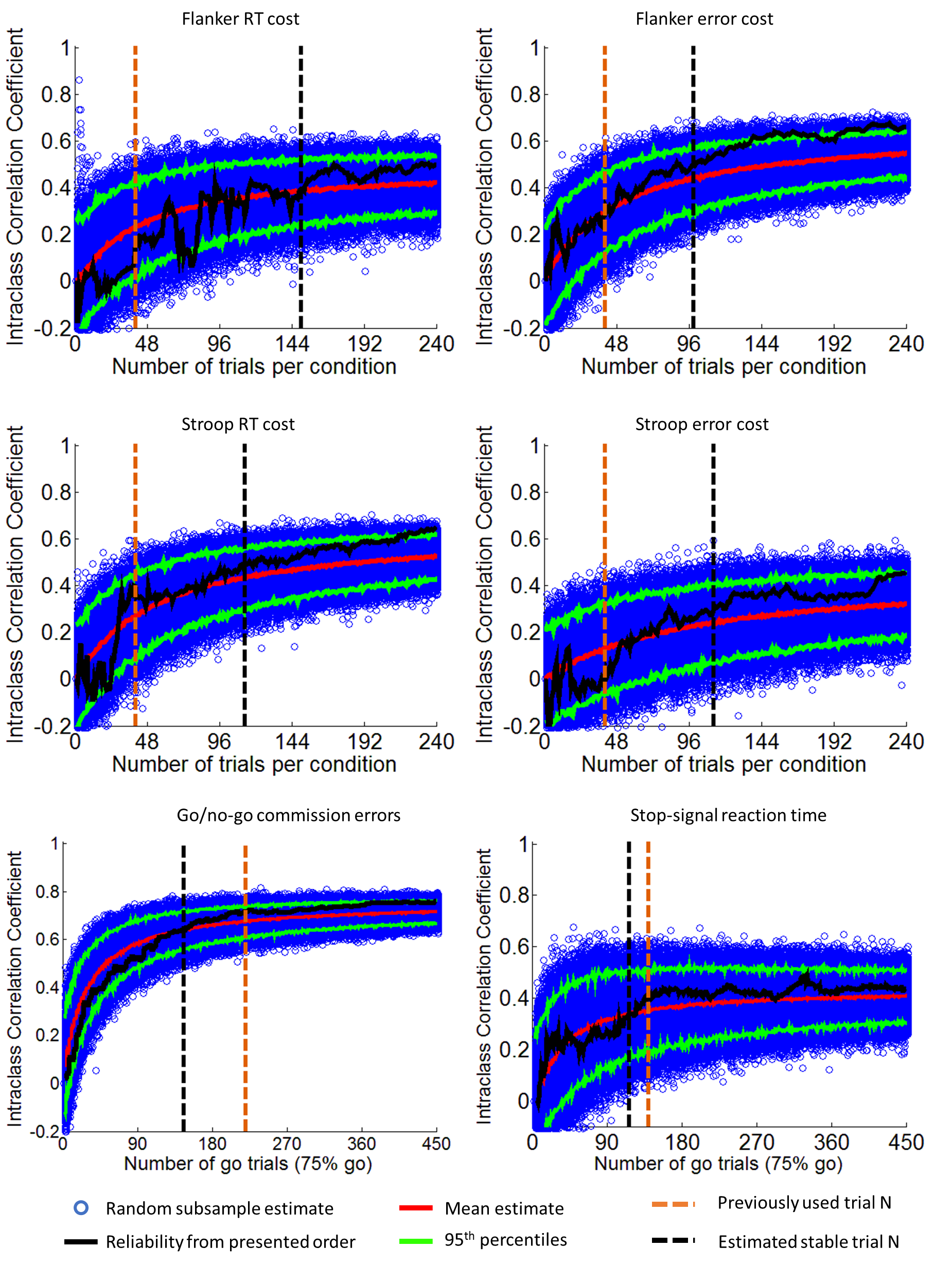


*Figure D1. The relationship between trial number per condition (x-axis) and reliability (ICC –y-axis; higher is better) for response control measures. Black solid lines represent reliability estimates at different trial numbers, using the order in which trials were completed by participants. Blue points represent reliability estimates from 1000 random subsamples (with replacement) for each trial number. The average ICC from these random subsamples is shown in red, with 2.75^th^ and 97.25^th^ percentiles in green. The black dashed line indicates where the true order provides a reliability estimate that consistently exceeds a threshold of .1 below the reliability estimate with all trials included. The number of trials used by previous reliability studies (see Table 1, where information was available; averaged where n>1) is indicated by the orange dashed line. Stop-signal reaction time is calculated using the integration method.*

The main points arising from this analysis are: First, for the Stroop and Flanker tasks, previous reliability studies have used fewer trials than our analysis indicates as sufficient for a stable estimate of reliability. At the number of trials used previously in the Flanker task (40 per condition), our subsampling estimates for the reliability of the RT and error costs ranged from negative values to .6; thus it is likely that previous reliability estimates (and any correlations with other tasks) based on such trial numbers are insecure. High trial numbers become especially important where there is low between-subject variance, since, as discussed above, when participants have similar levels of performance, small variation due to sampling can easily lead to changes in their rank ordering.

Second, note from the solid black lines that the estimates of reliability appear to plateau within the number of trials administered. This indicates that we would not see large gains in reliability for running more trials than we did. Third, for small trial numbers in most measures, the estimates from our ordered data fall below the average of our subsampled estimates (the black line is below the red line at first). This likely reflects that we did not administer practice trials in our studies, and these initial low reliability estimates reflect confounds of individual differences in learning rates. To verify this, we repeated the subsampling procedure while excluding the first block of trials from both sessions (see Supplementary material C). Though the tendency for the true order to be below the subsampled average was less apparent, excluding the first block did not result in higher final reliability values, nor did it substantially alter our suggestions for how many trials should be administered. Finally, our estimate of a sufficient number of trials for the SST (116 go, 39 stop) falls slightly below previous recommendations of at least 50 stop trials, based on simulations (Verbruggen, Chambers, & Logan, 2013). Our low estimate is likely due to the poor reliability observed overall for SSRT, such that it plateaued at a relatively low level early on. For this reason, we suggest following Verbruggen et al.’s recommendations.

Two further plots are shown below. First, we repeat the subsampling analysis for the tasks used in Studies 1 and 2 with the first block of trials excluded (Figure D2). Note that the relationship between trial number and reliability (or the final level of reliability observed) does not differ with these trials excluded. Trial subsampling plots for the tasks used in Study 3 are also shown (Figure D3). We do not identify optimal trial numbers for the measures for which the reliability did not differ significantly from 0.

**
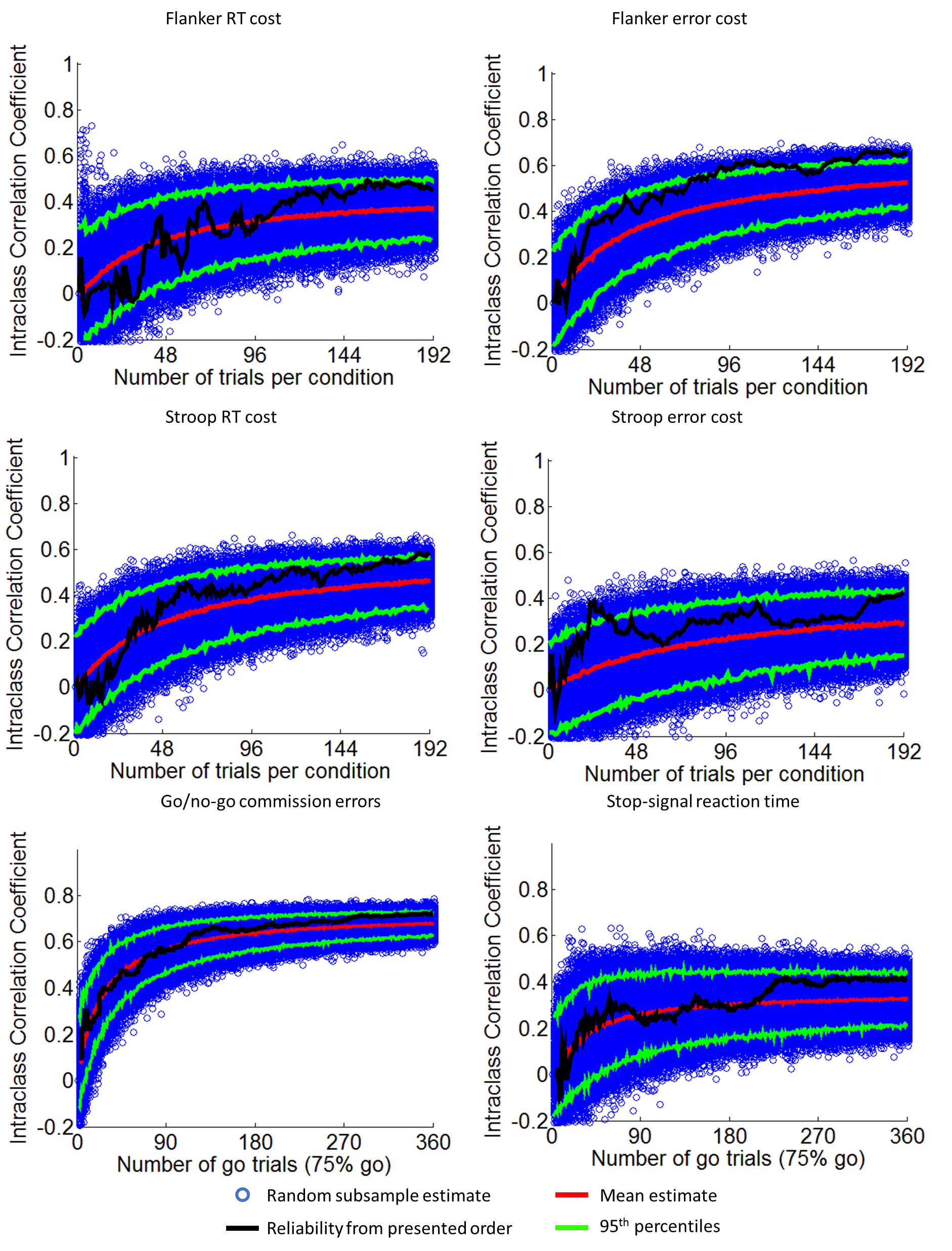
**

*Figure D2. Trial subsampling with first block removed from sessions 1 and 2.*


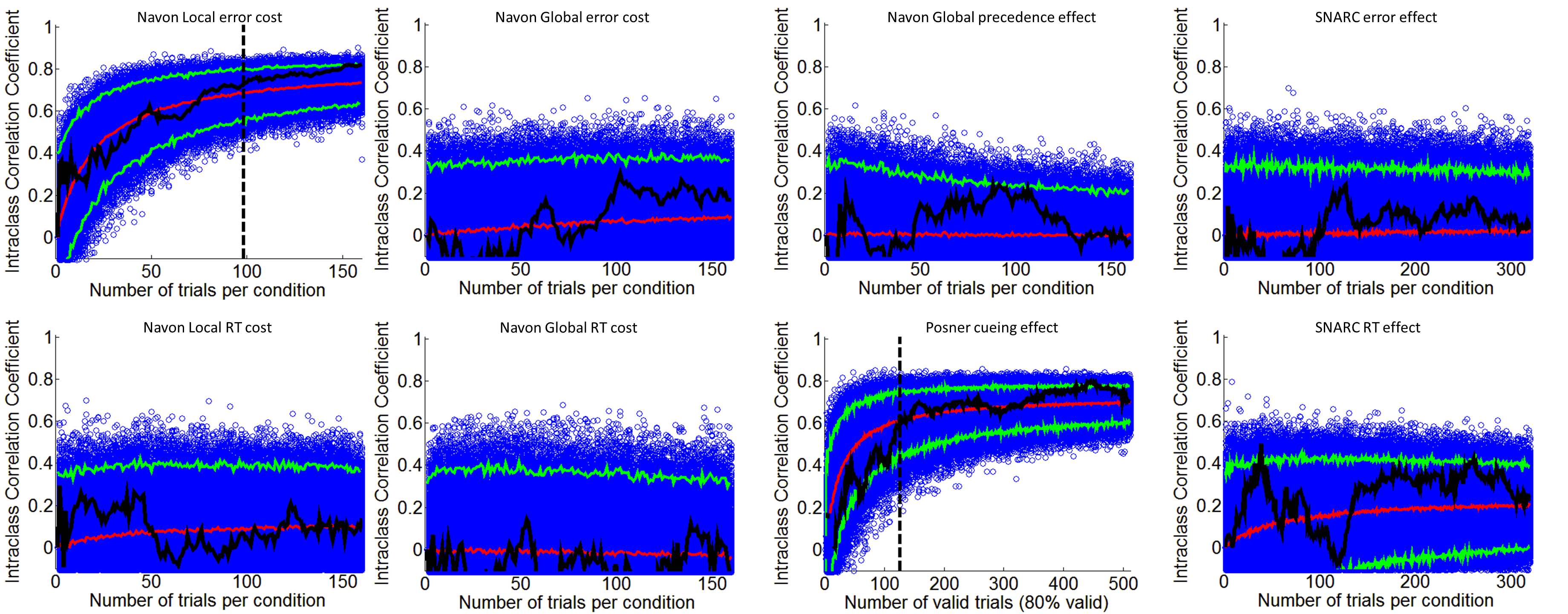


Figure D3. Trial subsampling plots for tasks administered in study 3.

**Supplementary material E – Variance component plots**

This section contains three plots of variance components, coinciding with the plots for the RT measures featured in the main text (Figure 2). First, figure E1 shows the relative variance components for the error measures in Studies 1-3. Figure E2 shows the absolute (non-normalised) variance components for the RT measures, and figure E3 shows the corresponding absolute variance components for error measures.


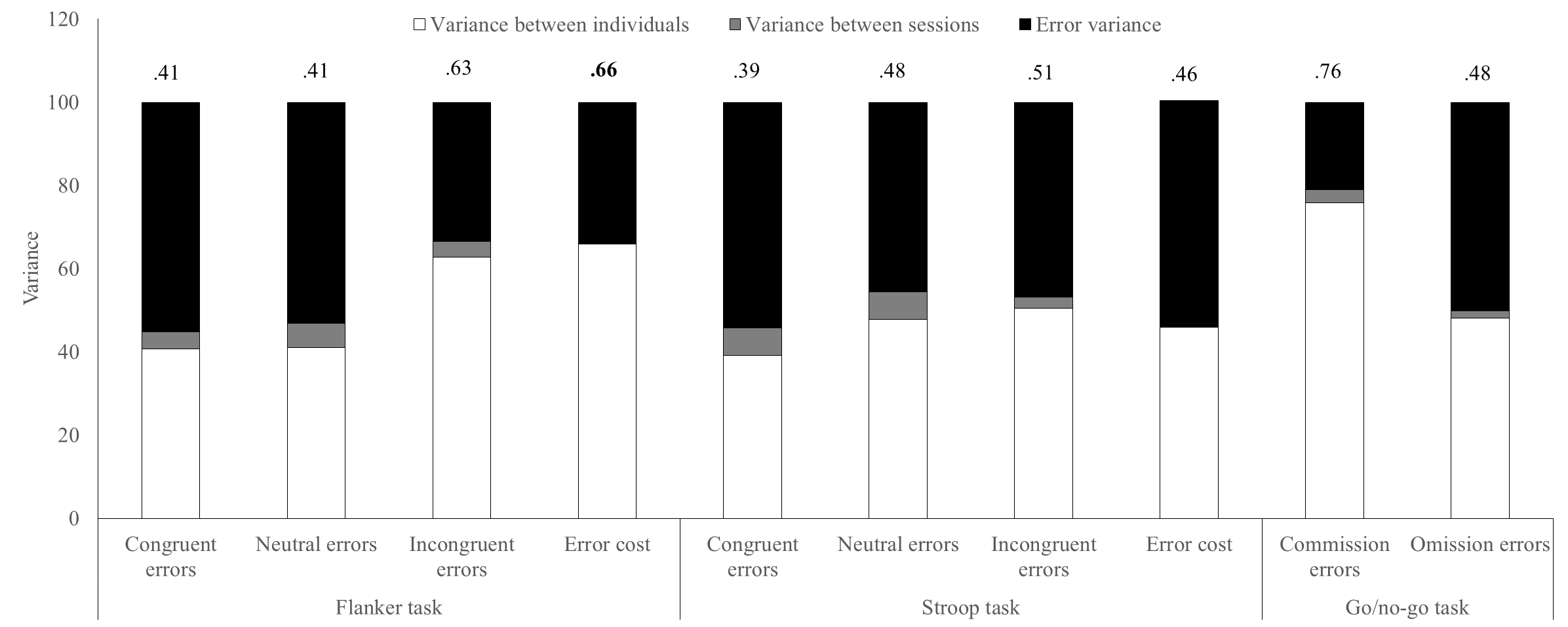


A


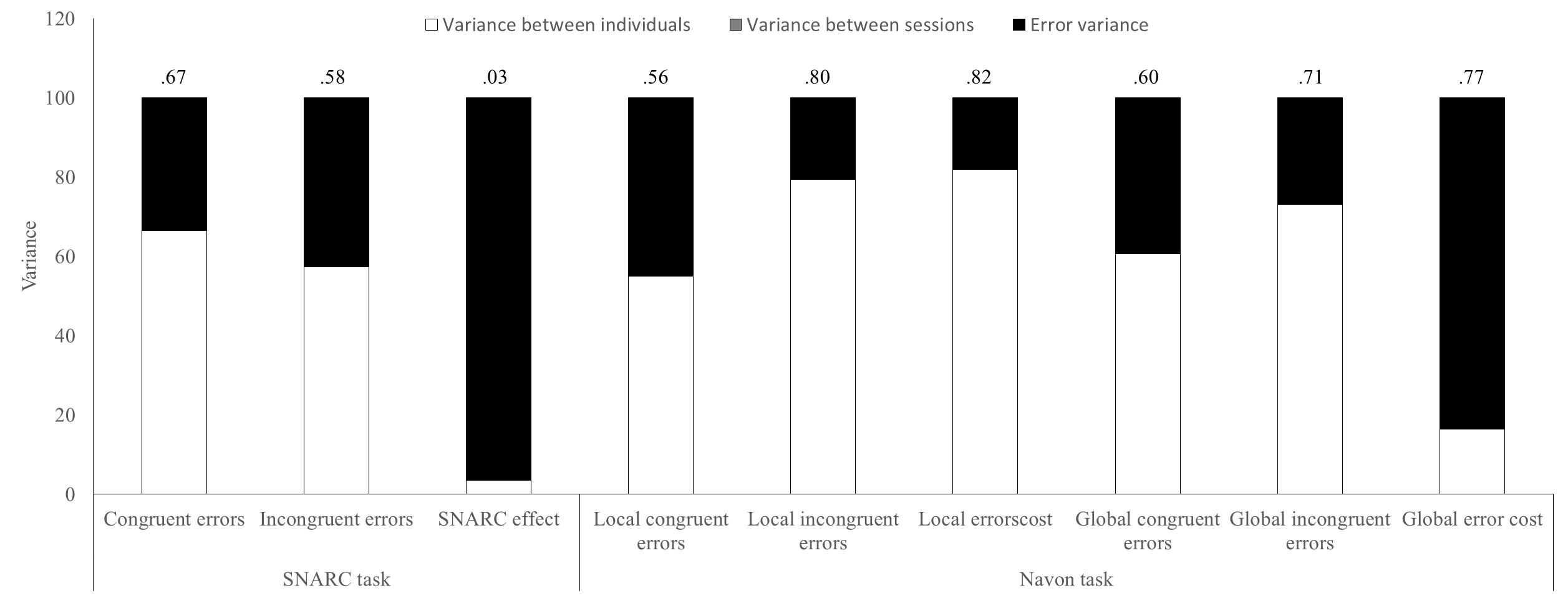


B

Figure E1. Relative size of variance components for error measures Studies 1 and 2 (A: Total Ns=99-104) and Study 3 (B: N=40). The size of the bar is normalised for the total amount of variance in the measure (see supplementary material B), and subdivided into variance accounted for by differences between participants (white), variance accounted for by differences between sessions (e.g. practice effects, grey), and error variance (black). The ICC reflects the proportion of the total variance attributed to variance between individuals, and is printed above each bar.


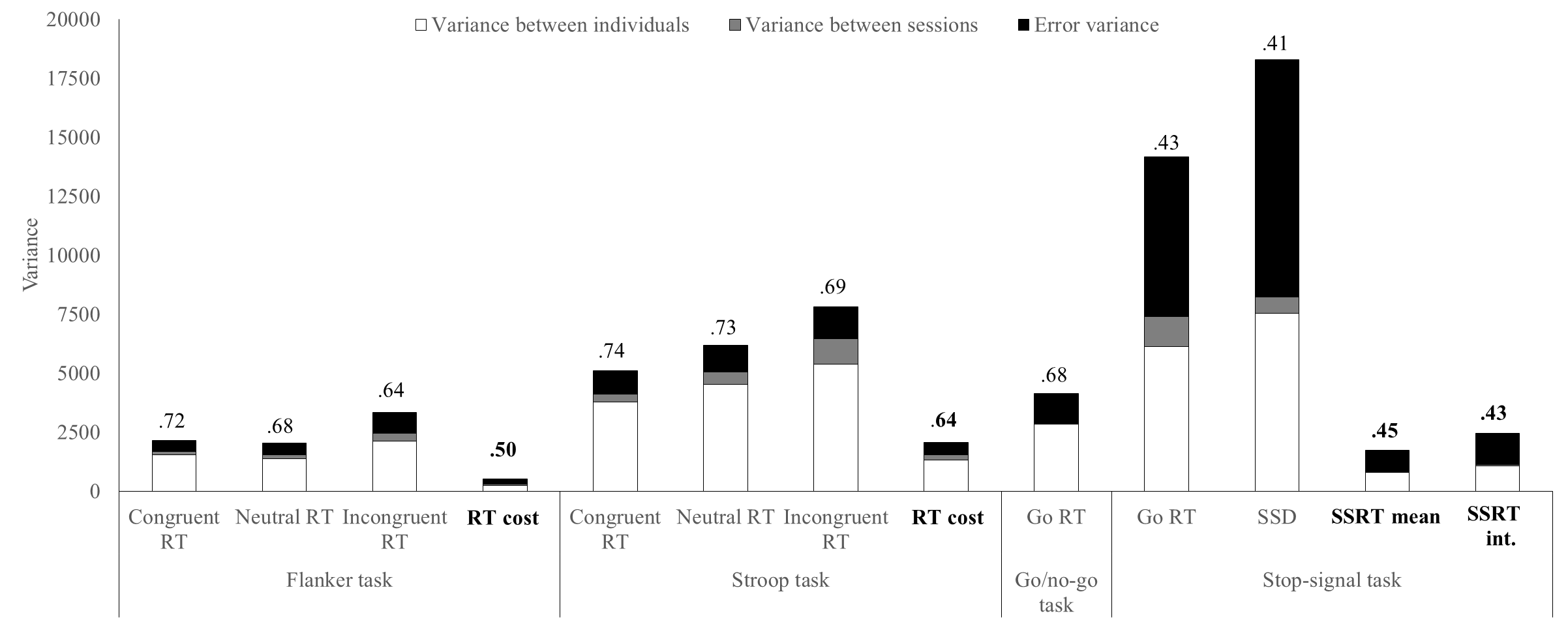

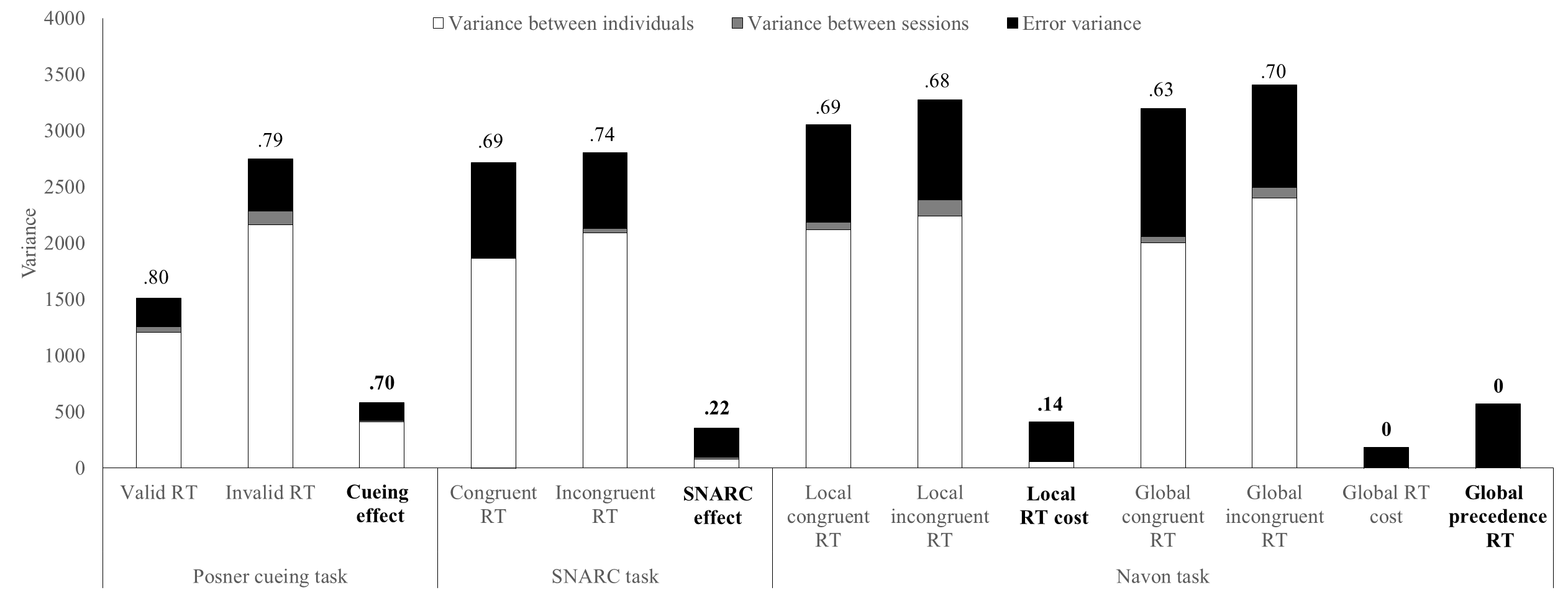


A

B

Figure E2. Size of variance components for RT measures on Study 1 and 2 (A:Ns = 99-104; see data analysis subsection) and Study 3 (B: N=40). The size of the bar reflects the total amount of variance in the measure, comprised of variance accounted for by differences between participants (white), variance accounted for by differences between sessions (e.g. practice effects, grey), and error variance (black). The ICC reflects the proportion of the total variance attributed to variance between individuals, and is printed above each bar. Note that the y-axis differs between A and B.

**
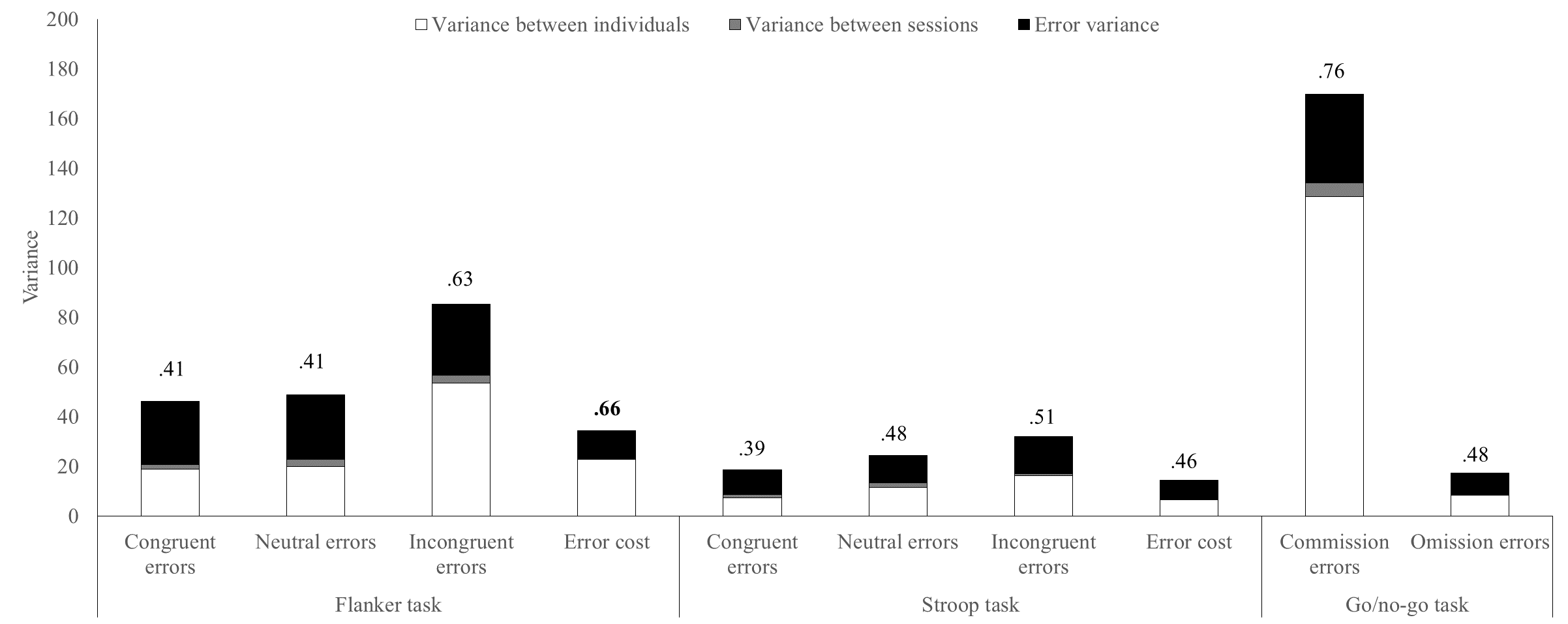
**

A

**
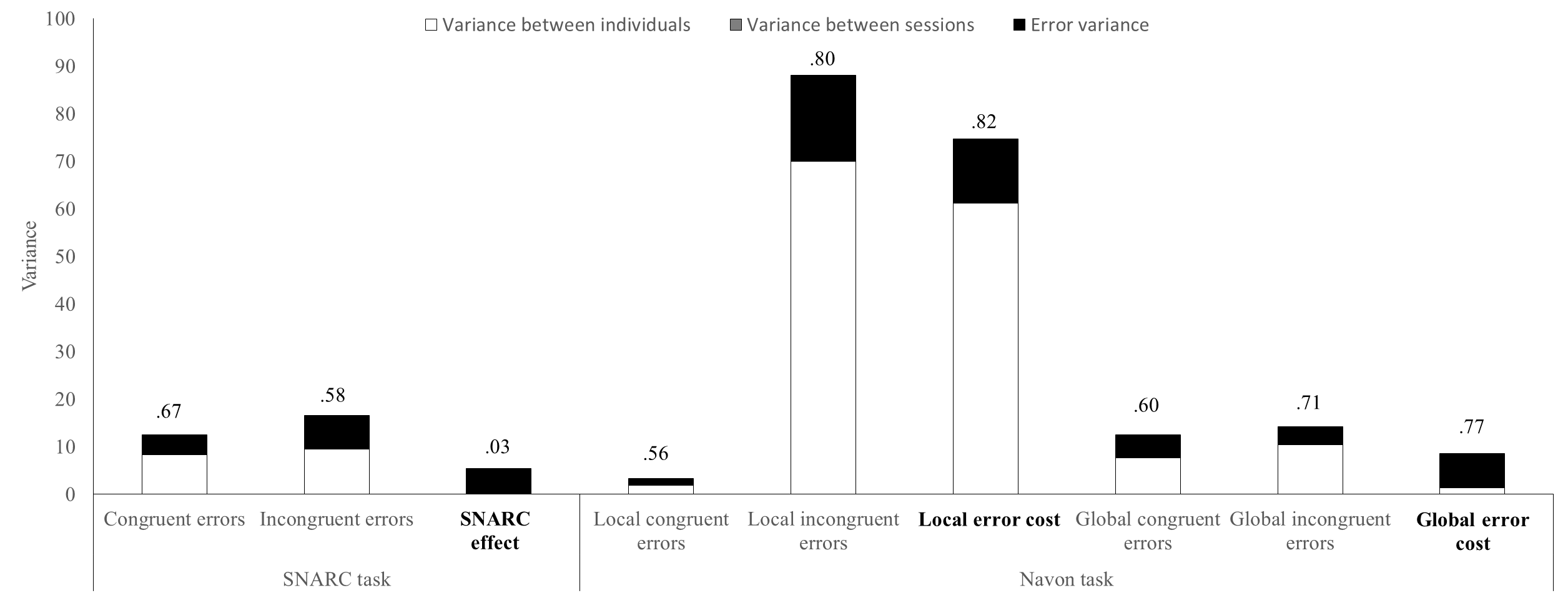
**

B

Figure E3. Size of variance components for error measures on Study 1 and 2 (A:Ns = 99-104; see data analysis subsection) and Study 3 (B: N=40). The size of the bar reflects the total amount of variance in the measure, comprised of variance accounted for by differences between participants (white), variance accounted for by differences between sessions (e.g. practice effects, grey), and error variance (black). The ICC reflects the proportion of the total variance attributed to variance between individuals, and is printed above each bar. Note that the y-axis differs between A and B.

**Supplementary Material F**

The tables below show the raw and disattenuated Spearman’s Rho correlations for the tasks used in Study 3. **Note that these disattenuated coefficients should be interpreted with caution**. Where ICCs are very low, as several of the measure in Study 3 were, the disattenuation formula inflates the correlations to an extent that is unlikely to be meaningful. We do not highlight ‘significant’ values for this reason. Coefficients that exceed 1/-1 after correction are reported as 1/-1. The formula cannot be calculated for measures with observed ICCs of 0, so these cells are blank.

**Raw correlations (Study 3)**

|  | Posner effect | SNARC RT | SNARC Error | Navon Local RT | Navon Local Error | Navon Global RT | Navon Global Error |
| --- | --- | --- | --- | --- | --- | --- | --- |
| Posner effect |  |  |  |  |  |  |  |
| SNARC RT effect | 0.14 |  |  |  |  |  |  |
| SNARC Error effect | -0.08 | 0.20 |  |  |  |  |  |
| Navon Local RT cost | 0.12 | -0.09 | 0.08 |  |  |  |  |
| Navon Local Error cost | -0.06 | 0.05 | -0.05 | 0.32 |  |  |  |
| Navon Global RT cost | 0.19 | -0.05 | -0.08 | 0.25 | 0.10 |  |  |
| Navon Global Error cost | 0.22 | 0.14 | -0.24 | 0.17 | 0.22 | 0.03 |  |
| Navon Global Precedence | -0.12 | -0.20 | 0.21 | -0.03 | -0.17 | -0.23 | -0.28 |

**Disattenuated correlations (Study 3)**

|  | Posner effect | SNARC RT | SNARC Error | Navon Local RT | Navon Local Error | Navon Global RT | Navon Global Error | Navon Global Prec. |
| --- | --- | --- | --- | --- | --- | --- | --- | --- |
| Posner effect |  |  |  |  |  |  |  |  |
| SNARC RT effect | 0.36 |  |  |  |  |  |  |  |
| SNARC Error effect | -0.55 | 1 |  |  |  |  |  |  |
| Navon Local RT cost | 0.38 | -0.49 | 1 |  |  |  |  |  |
| Navon Local Error cost | -0.08 | 0.12 | -0.29 | 0.94 |  |  |  |  |
| Navon Global RT cost |  |  |  |  |  |  |  |  |
| Navon Global Error cost | 0.62 | 0.74 | 1 | 1 | 0.59 |  |  |  |
| Navon Global Precedence |  |  |  |  |  |  |  |  |

**Supplementary material G**

This section contains the within session reliabilities for studies 1-3. Tables G1 and G2 show the ICCs for absolute agreement calculated on the first and second half of each session for Studies 1 and 2 (G1) and Study 3 (G2). Tables G3 and G4 show the ICCs ‘stepped up’ using the Spearman-Brown prophecy formula. The Spearman-Brown formula is used to estimate the reliability of a test if the length of the test is increased. In other words, it uses the correlation between the two halves to estimate the reliability of the full dataset. Note that this assumes the two halves constitute parallel forms. Tables G5 and G6 show the within-session reliabilities when comparing odd versus even numbered trials. Note that these are generally the highest of our estimates, though many are still sub-optimal for the key measures. The ICCs for the stop-signal task are notably high in Table G5. In part, this likely reflects the tracking procedure used, as the SSD on consecutive stop trials will necessarily be ±50ms from each other.

Table G1. ICCs for absolute agreement (95% confidence intervals in parentheses) calculated on the first and second half of the data for each session for Studies 1 and 2.

| Task | Measure | Study 1 | | Study 2 | |
| --- | --- | --- | --- | --- | --- |
|  |  | Session 1 | Session 2 | Session 1 | Session 2 |
| Flanker task | Congruent RT | 0.83 (0.72-0.9) | 0.91 (0.84-0.95) | 0.9 (0.84-0.94) | 0.5 (0.28-0.67) |
|  | Neutral RT | 0.85 (0.74-0.91) | 0.94 (0.84-0.97) | 0.9 (0.84-0.94) | 0.26 (0-0.48) |
|  | Incongruent RT | 0.81 (0.65-0.9) | 0.93 (0.88-0.96) | 0.88 (0.63-0.95) | 0.37 (0.13-0.57) |
|  | **RT cost** | **0.45 (0.09-0.69)** | **0.4 (0.12-0.61)** | **0.64 (0.23-0.82)** | **0.29 (0.04-0.51)** |
|  |  |  |  |  |  |
|  | Congruent errors | 0.18 (-0.11-0.44) | 0.86 (0.72-0.93) | 0.72 (0.52-0.84) | 0.86 (0.76-0.91) |
|  | Neutral errors | 0.12 (-0.16-0.39) | 0.86 (0.75-0.92) | 0.68 (0.33-0.84) | 0.79 (0.55-0.89) |
|  | Incongruent errors | 0.44 (0.18-0.64) | 0.85 (0.74-0.91) | 0.81 (0.7-0.89) | 0.83 (0.71-0.9) |
|  | **Error cost** | **0.55 (0.31-0.72)** | **0.59 (0.36-0.75)** | **0.65 (0.46-0.78)** | **0.65 (0.47-0.77)** |
| Stroop task | Congruent RT | 0.79 (0.66-0.88) | 0.9 (0.84-0.95) | 0.83 (0.73-0.9) | 0.81 (0.7-0.89) |
|  | Neutral RT | 0.82 (0.71-0.9) | 0.9 (0.83-0.94) | 0.85 (0.75-0.91) | 0.85 (0.75-0.91) |
|  | Incongruent RT | 0.85 (0.74-0.92) | 0.87 (0.78-0.93) | 0.8 (0.67-0.88) | 0.76 (0.62-0.85) |
|  | **RT cost** | **0.57 (0.34-0.74)** | **0.46 (0.2-0.66)** | **0.59 (0.38-0.74)** | **0.57 (0.36-0.73)** |
|  |  |  |  |  |  |
|  | Congruent errors | 0.59 (0.25-0.78) | 0.78 (0.63-0.87) | 0.5 (0.27-0.67) | 0.43 (0.19-0.62) |
|  | Neutral errors | 0.52 (0.25-0.71) | 0.82 (0.68-0.9) | 0.6 (0.4-0.74) | 0.49 (0.27-0.67) |
|  | Incongruent errors | 0.7 (0.52-0.82) | 0.89 (0.81-0.93) | 0.54 (0.33-0.71) | 0.53 (0.31-0.69) |
|  | **Error cost** | **0.5 (0.2-0.7)** | **0.6 (0.37-0.76)** | **0.32 (0.07-0.54)** | **0.24 (-0.03-0.47)** |
| Go/No-go task | Go RT | 0.70 (0.51-0.82) | 0.86 (0.71-0.93) | 0.81 (0.66-0.89) | 0.75 (0.56-0.86) |
|  | **Commission errors** | **0.84 (0.72-0.91)** | **0.78 (0.64-0.87)** | **0.75 (0.49-0.87)** | **0.77 (0.6-0.87)** |
|  | Omission errors | 0.26 (-0.02-0.5) | 0.7 (0.51-0.82) | 0.25 (-0.02-0.48) | 0.68 (0.51-0.8) |
| Stop-signal task | Go RT | 0.95 (0.90-0.98) | 0.87 (0.77-0.93) | 0.85 (0.73-0.91) | 0.92 (0.85-0.96) |
|  | Mean SSD | 0.9 (0.76-0.95) | 0.87 (0.78-0.93) | 0.82 (0.67-0.9) | 0.91 (0.84-0.95) |
|  | **SSRT mean** | **0.48 (0.23-0.68)** | **0.68 (0.48-0.81)** | **0.54 (0.32-0.7)** | **0.66 (0.48-0.79)** |
|  | **SSRT integration** | **0.52 (0.27-0.7)** | **0.63 (0.42-0.78)** | **0.57 (0.35-0.72)** | **0.75 (0.61-0.85)** |

Table G2. ICCs for absolute agreement (95% confidence intervals in parentheses) calculated on the first and second half of the data for each session for Study 3.

|  | Measure | Session 1 | Session 2 |
| --- | --- | --- | --- |
| Posner task | Valid RT | 0.8 (0.66-0.89) | 0.81 (0.67-0.9) |
|  | Invalid RT | 0.73 (0.54-0.85) | 0.86 (0.76-0.92) |
|  | **Cueing effect** | **0.65 (0.43-0.8)** | **0.7 (0.51-0.83)** |
| SNARC task | Congruent RT | 0.74 (0.57-0.85) | 0.81 (0.67-0.89) |
|  | Incongruent RT | 0.73 (0.54-0.84) | 0.92 (0.86-0.96) |
|  | **SNARC effect RT** | **-0.37 (-0.61--0.06)** | **-0.33 (-0.59--0.02)** |
|  |  |  |  |
|  | Congruent errors | 0.82 (0.69-0.9) | 0.55 (0.3-0.73) |
|  | Incongruent errors | 0.61 (0.37-0.77) | 0.83 (0.7-0.91) |
|  | **SNARC effect errors** | **0.02 (-0.3-0.33)** | **0.2 (-0.12-0.48)** |
| Navon task | Local congruent RT | 0.8 (0.61-0.9) | 0.91 (0.84-0.95) |
|  | Local incongruent RT | 0.75 (0.51-0.87) | 0.86 (0.75-0.92) |
|  | **Local RT cost** | **0.45 (0.17-0.66)** | **0.16 (-0.16-0.44)** |
|  |  |  |  |
|  | Local congruent errors | 0.37 (0.07-0.61) | 0.38 (0.08-0.62) |
|  | Local incongruent errors | 0.78 (0.63-0.88) | 0.83 (0.7-0.9) |
|  | **Local error cost** | **0.68 (0.47-0.82)** | **0.74 (0.56-0.85)** |
|  |  |  |  |
|  | Global congruent RT | 0.87 (0.76-0.93) | 0.87 (0.77-0.93) |
|  | Global incongruent RT | 0.84 (0.71-0.91) | 0.81 (0.66-0.89) |
|  | **Global RT cost** | **0.19 (-0.12-0.46)** | **0.13 (-0.18-0.42)** |
|  |  |  |  |
|  | Global congruent errors | 0.61 (0.38-0.78) | 0.52 (0.26-0.72) |
|  | Global incongruent errors | 0.8 (0.66-0.89) | 0.51 (0.24-0.7) |
|  | **Global error cost** | **0.28 (-0.03-0.54)** | **-0.03 (-0.34-0.29)** |
|  |  |  |  |
|  | **Global precedence effect (RT)** | **-0.12 (-0.38-0.18)** | **0.38 (0.08-0.61)** |

Table G3. ICCs for first and second half of the data for each session for Studies 1 and 2 stepped up with Spearman-Brown prophecy formula.

| Task | Measure | Study 1 | | Study 2 | |
| --- | --- | --- | --- | --- | --- |
|  |  | Session 1 | Session 2 | Session 1 | Session 2 |
| Flanker task | Congruent RT | 0.91 | 0.95 | 0.95 | 0.67 |
|  | Neutral RT | 0.92 | 0.97 | 0.95 | 0.41 |
|  | Incongruent RT | 0.9 | 0.96 | 0.94 | 0.54 |
|  | **RT cost** | **0.62** | **0.57** | **0.78** | **0.45** |
|  |  |  |  |  |  |
|  | Congruent errors | 0.31 | 0.92 | 0.84 | 0.92 |
|  | Neutral errors | 0.21 | 0.92 | 0.81 | 0.88 |
|  | Incongruent errors | 0.61 | 0.92 | 0.9 | 0.91 |
|  | **Error cost** | **0.71** | **0.74** | **0.79** | **0.79** |
| Stroop task | Congruent RT | 0.88 | 0.95 | 0.91 | 0.9 |
|  | Neutral RT | 0.9 | 0.95 | 0.92 | 0.92 |
|  | Incongruent RT | 0.92 | 0.93 | 0.89 | 0.86 |
|  | **RT cost** | **0.73** | **0.63** | **0.74** | **0.73** |
|  |  |  |  |  |  |
|  | Congruent errors | 0.74 | 0.88 | 0.67 | 0.6 |
|  | Neutral errors | 0.68 | 0.9 | 0.75 | 0.66 |
|  | Incongruent errors | 0.82 | 0.94 | 0.7 | 0.69 |
|  | **Error cost** | **0.67** | **0.75** | **0.48** | **0.39** |
| Go/No-go task | Go RT | 0.82 | 0.92 | 0.89 | 0.86 |
|  | **Commission errors** | **0.91** | **0.88** | **0.86** | **0.87** |
|  | Omission errors | 0.41 | 0.82 | 0.4 | 0.81 |
| Stop-signal task | Go RT | 0.97 | 0.93 | 0.92 | 0.96 |
|  | Mean SSD | 0.95 | 0.93 | 0.9 | 0.95 |
|  | **SSRT mean** | **0.65** | **0.81** | **0.7** | **0.8** |
|  | **SSRT integration** | **0.68** | **0.77** | **0.73** | **0.86** |

Table G4. ICCs for first and second half of the data for each session for Study 3 stepped up with Spearman-Brown prophecy formula.

|  | Measure | Session 1 | Session 2 |
| --- | --- | --- | --- |
| Posner task | Valid RT | 0.89 | 0.9 |
|  | Invalid RT | 0.84 | 0.92 |
|  | **Cueing effect** | **0.79** | **0.82** |
| SNARC task | Congruent RT | 0.85 | 0.9 |
|  | Incongruent RT | 0.84 | 0.96 |
|  | **SNARC effect RT** | **0** | **0** |
|  |  |  |  |
|  | Congruent errors | 0.9 | 0.71 |
|  | Incongruent errors | 0.76 | 0.91 |
|  | **SNARC effect errors** | **0.04** | **0.33** |
| Navon task | Local congruent RT | 0.89 | 0.95 |
|  | Local incongruent RT | 0.86 | 0.92 |
|  | **Local RT cost** | **0.62** | **0.28** |
|  |  |  |  |
|  | Local congruent errors | 0.54 | 0.55 |
|  | Local incongruent errors | 0.88 | 0.91 |
|  | **Local error cost** | **0.81** | **0.85** |
|  |  |  |  |
|  | Global congruent RT | 0.93 | 0.93 |
|  | Global incongruent RT | 0.91 | 0.9 |
|  | **Global RT cost** | **0.32** | **0.23** |
|  |  |  |  |
|  | Global congruent errors | 0.76 | 0.68 |
|  | Global incongruent errors | 0.89 | 0.68 |
|  | **Global error cost** | **0.44** | **-0.06** |
|  |  |  |  |
|  | **Global precedence effect (RT)** | **0** | **0.55** |

Table G5. ICCs for absolute agreement (95% confidence intervals in parentheses) calculated on odd and even trials from the data for each session for Studies 1 and 2.

| Task | Measure | Study 1 | | Study 2 | |
| --- | --- | --- | --- | --- | --- |
|  |  | Session 1 | Session 2 | Session 1 | Session 2 |
| Flanker task | Congruent RT | 0.98 (0.96-0.99) | 0.99 (0.99-1) | 0.97 (0.95-0.98) | 0.93 (0.89-0.96) |
|  | Neutral RT | 0.97 (0.94-0.98) | 0.99 (0.98-0.99) | 0.97 (0.96-0.98) | 0.93 (0.89-0.96) |
|  | Incongruent RT | 0.97 (0.95-0.98) | 0.96 (0.93-0.98) | 0.97 (0.95-0.98) | 0.95 (0.92-0.97) |
|  | **RT cost** | **0.67 (0.48-0.8)** | **0.62 (0.41-0.77)** | **0.8 (0.68-0.88)** | **0.55 (0.34-0.71)** |
|  |  |  |  |  |  |
|  | Congruent errors | 0.89 (0.81-0.94) | 0.86 (0.76-0.92) | 0.8 (0.69-0.88) | 0.95 (0.92-0.97) |
|  | Neutral errors | 0.87 (0.77-0.92) | 0.89 (0.81-0.94) | 0.85 (0.76-0.91) | 0.96 (0.93-0.97) |
|  | Incongruent errors | 0.87 (0.78-0.93) | 0.9 (0.83-0.94) | 0.86 (0.78-0.92) | 0.85 (0.76-0.91) |
|  | **Error cost** | **0.53 (0.29-0.71)** | **0.64 (0.44-0.78)** | **0.68 (0.51-0.8)** | **0.55 (0.34-0.71)** |
| Stroop task | Congruent RT | 0.94 (0.89-0.97) | 0.98 (0.96-0.99) | 0.93 (0.89-0.96) | 0.94 (0.89-0.96) |
|  | Neutral RT | 0.96 (0.93-0.98) | 0.91 (0.84-0.95) | 0.94 (0.9-0.97) | 0.91 (0.85-0.95) |
|  | Incongruent RT | 0.95 (0.92-0.97) | 0.95 (0.91-0.97) | 0.94 (0.91-0.97) | 0.91 (0.85-0.94) |
|  | **RT cost** | **0.61 (0.39-0.76)** | **0.6 (0.38-0.75)** | **0.72 (0.57-0.83)** | **0.67 (0.5-0.79)** |
|  |  |  |  |  |  |
|  | Congruent errors | 0.68 (0.49-0.81) | 0.84 (0.73-0.91) | 0.63 (0.44-0.77) | 0.77 (0.64-0.86) |
|  | Neutral errors | 0.64 (0.43-0.78) | 0.73 (0.56-0.84) | 0.79 (0.67-0.87) | 0.75 (0.61-0.85) |
|  | Incongruent errors | 0.74 (0.58-0.85) | 0.87 (0.78-0.93) | 0.74 (0.6-0.84) | 0.82 (0.72-0.89) |
|  | **Error cost** | **0.44 (0.18-0.64)** | **0.52 (0.27-0.7)** | **0.25 (-0.01-0.48)** | **0.36 (0.12-0.56)** |
| Go/No-go task | Go RT | 0.99 (0.98-0.99) | 0.99 (0.99-1) | 0.98 (0.97-0.99) | 0.99 (0.99-1) |
|  | **Commission errors** | **0.84 (0.72-0.91)** | **0.86 (0.76-0.92)** | **0.85 (0.72-0.91)** | **0.91 (0.86-0.95)** |
|  | Omission errors | 0.93 (0.88-0.96) | 0.97 (0.94-0.98) | 0.97 (0.96-0.98) | 0.98 (0.97-0.99) |
| Stop-signal task | Go RT | 1 (0.99-1) | 0.99 (0.98-1) | 1 (0.99-1) | 0.99 (0.98-0.99) |
|  | Mean SSD | 1 (0.99-1) | 0.99 (0.99-1) | 0.99 (0.99-1) | 0.99 (0.99-1) |
|  | **SSRT mean** | **0.91 (0.84-0.95)** | **0.90 (0.83-0.94)** | **0.9 (0.84-0.94)** | **0.89 (0.82-0.93)** |
|  | **SSRT integration** | **0.84 (0.73-0.91)** | **0.88 (0.79-0.93)** | **0.82 (0.71-0.89)** | **0.89 (0.82-0.93)** |

Table G6. ICCs for absolute agreement (95% confidence intervals in parentheses) calculated from odd and even trials for each session for Study 3.

|  | Measure | Session 1 | Session 2 |
| --- | --- | --- | --- |
| Posner task | Valid RT | 0.99 (0.98-0.99) | 0.98 (0.96-0.99) |
|  | Invalid RT | 0.95 (0.91-0.98) | 0.96 (0.93-0.98) |
|  | **Cueing effect** | **0.78 (0.62-0.87)** | **0.78 (0.63-0.88)** |
| SNARC task | Congruent RT | 0.98 (0.96-0.99) | 0.98 (0.97-0.99) |
|  | Incongruent RT | 0.98 (0.96-0.99) | 0.98 (0.96-0.99) |
|  | **SNARC effect RT** | **0.74 (0.56-0.85)** | **0.66 (0.44-0.8)** |
|  |  |  |  |
|  | Congruent errors | 0.69 (0.49-0.82) | 0.73 (0.54-0.85) |
|  | Incongruent errors | 0.72 (0.53-0.84) | 0.67 (0.45-0.81) |
|  | **SNARC effect errors** | **-0.06 (-0.36-0.24)** | **0.12 (-0.2-0.42)** |
| Navon task | Local congruent RT | 0.97 (0.95-0.98) | 0.95 (0.9-0.97) |
|  | Local incongruent RT | 0.95 (0.91-0.97) | 0.95 (0.91-0.97) |
|  | **Local RT cost** | **0.54 (0.28-0.73)** | **0.41 (0.11-0.64)** |
|  |  |  |  |
|  | Local congruent errors | 0.33 (0.03-0.58) | 0.23 (-0.06-0.49) |
|  | Local incongruent errors | 0.87 (0.76-0.93) | 0.9 (0.82-0.95) |
|  | **Local error cost** | **0.8 (0.65-0.89)** | **0.79 (0.63-0.88)** |
|  |  |  |  |
|  | Global congruent RT | 0.96 (0.93-0.98) | 0.97 (0.94-0.98) |
|  | Global incongruent RT | 0.97 (0.94-0.98) | 0.95 (0.91-0.97) |
|  | **Global RT cost** | **-0.11 (-0.41-0.21)** | **0.33 (0.02-0.58)** |
|  |  |  |  |
|  | Global congruent errors | 0.54 (0.28-0.72) | 0.47 (0.19-0.68) |
|  | Global incongruent errors | 0.52 (0.25-0.71) | 0.47 (0.19-0.68) |
|  | **Global error cost** | **-0.18 (-0.47-0.14)** | **0 (-0.32-0.31)** |
|  |  |  |  |
|  | **Global precedence effect (RT)** | **0.6 (0.36-0.77)** | **0.68 (0.46-0.81)** |
